# GeneCompass: deciphering universal gene regulatory mechanisms with a knowledge-informed cross-species foundation model

**DOI:** 10.1038/s41422-024-01034-y

**Published:** 2024-10-08

**Authors:** Xiaodong Yang, Guole Liu, Guihai Feng, Dechao Bu, Pengfei Wang, Jie Jiang, Shubai Chen, Qinmeng Yang, Hefan Miao, Yiyang Zhang, Zhenpeng Man, Zhongming Liang, Zichen Wang, Yaning Li, Zheng Li, Yana Liu, Yao Tian, Wenhao Liu, Cong Li, Ao Li, Jingxi Dong, Zhilong Hu, Chen Fang, Lina Cui, Zixu Deng, Haiping Jiang, Wentao Cui, Jiahao Zhang, Zhaohui Yang, Handong Li, Xingjian He, Liqun Zhong, Jiaheng Zhou, Zijian Wang, Qingqing Long, Ping Xu, The X-Compass Consortium, Jie Jiang, Qinmeng Yang, Zheng Li, Xingjian He, Zijian Wang, Qingqing Long, Xin Li, Hongmei Wang, Baoyang Hu, Wei Li, Fei Gao, Jingtao Guo, Leqian Yu, Qi Gu, Weiwei Zhai, Zhengting Zou, Guihai Feng, Wenhao Liu, Yao Tian, Chen Fang, Jingxi Dong, Yana Liu, Jingqi Yu, Wenhui Wu, Xinxin Lin, Cong Li, Yu Zou, Yongshun Ren, Fan Li, Yixiao Zhao, Yike Xin, Longfei Han, Shuyang Jiang, Kai Ma, Qicheng Chen, Haoyuan Wang, Huanhuan Wu, Chaofan He, Yilong Hu, Shuyu Guo, Yiyun Li, Yuanchun Zhou, Yangang Wang, Xuezhi Wang, Pengfei Wang, Fei Li, Zhen Meng, Zaitian Wang, Ping Xu, Wentao Cui, Zhilong Hu, Huimin He, Shan Zong, Jiajia Wang, Yan Chen, Chunyang Zhang, Chengrui Wang, Ran Zhang, Meng Xiao, Yining Wang, Yiqiang Chen, Yi Zhao, Xiaodong Yang, Dechao Bu, Xin Qin, Jiaxin Qin, Zhaohui Yang, Chenhao Li, Zhufeng Xu, Zeyuan Zhang, Xiaoning Qi, Shubai Chen, Wuliang Huang, Yaning Li, Ge Yang, Jing Liu, Guole Liu, Liqun Zhong, Yaoru Luo, Jiaheng Zhou, Zichen Wang, Qinxuan Luo, Ziwen Liu, Ao Li, Teng Wang, Yiming Huang, Handong Li, Yong Wang, Shihua Zhang, Jiahao Zhang, Yiyang Zhang, Shirui Li, Zhongming Liang, Zhenpeng Man, Kangning Dong, Qunlun Shen, Hongmei Wang, Zhen Meng, Xuezhi Wang, Yangang Wang, Yong Wang, Shihua Zhang, Jingtao Guo, Yi Zhao, Yuanchun Zhou, Fei Li, Jing Liu, Yiqiang Chen, Ge Yang, Xin Li

**Affiliations:** 1 State Key Laboratory of Stem Cell and Reproductive Biology, Institute of Zoology, Chinese Academy of Sciences, Beijing, China; 2 Beijing Key Laboratory of Mobile Computing and Pervasive Device, Institute of Computing Technology, Chinese Academy of Sciences, Beijing, China; 3 University of Chinese Academy of Sciences, Beijing, China; 4 State Key Laboratory of Multimodal Artificial Intelligence Systems, Institute of Automation, Chinese Academy of Sciences, Beijing, China; 5 School of Artificial Intelligence, University of Chinese Academy of Sciences, Beijing, China; 6 Institute for Stem Cell and Regenerative Medicine, Chinese Academy of Sciences, Beijing, China; 7 Beijing Institute for Stem Cell and Regenerative Medicine, Beijing, China; 8 Research Center for Ubiquitous Computing Systems, Institute of Computing Technology, Chinese Academy of Sciences, Beijing, China; 9 Computer Network Information Center, Chinese Academy of Sciences, Beijing, China; 10 Institute of Automation, Chinese Academy of Sciences, Beijing, China; 11 CEMS, NCMIS, HCMS, MDIS, RCSDS, Academy of Mathematics and Systems Science, Chinese Academy of Sciences, Beijing, China; 12 Institute of Zoology, Chinese Academy of Sciences, Beijing, China; 13 Institute of Computing Technology, Chinese Academy of Sciences, Beijing, China; 14 Academy of Mathematics and Systems Science, Chinese Academy of Sciences, Beijing, China

**Keywords:** Bioinformatics, Developmental biology

## Abstract

Deciphering universal gene regulatory mechanisms in diverse organisms holds great potential for advancing our knowledge of fundamental life processes and facilitating clinical applications. However, the traditional research paradigm primarily focuses on individual model organisms and does not integrate various cell types across species. Recent breakthroughs in single-cell sequencing and deep learning techniques present an unprecedented opportunity to address this challenge. In this study, we built an extensive dataset of over 120 million human and mouse single-cell transcriptomes. After data preprocessing, we obtained 101,768,420 single-cell transcriptomes and developed a knowledge-informed cross-species foundation model, named GeneCompass. During pre-training, GeneCompass effectively integrated four types of prior biological knowledge to enhance our understanding of gene regulatory mechanisms in a self-supervised manner. By fine-tuning for multiple downstream tasks, GeneCompass outperformed state-of-the-art models in diverse applications for a single species and unlocked new realms of cross-species biological investigations. We also employed GeneCompass to search for key factors associated with cell fate transition and showed that the predicted candidate genes could successfully induce the differentiation of human embryonic stem cells into the gonadal fate. Overall, GeneCompass demonstrates the advantages of using artificial intelligence technology to decipher universal gene regulatory mechanisms and shows tremendous potential for accelerating the discovery of critical cell fate regulators and candidate drug targets.

## Introduction

Vertebrate organisms are intricate systems composed of up to trillions of cells classified into hundreds of different types. These cells collaborate to form diverse tissues and organs, each with unique physiological functions.^1,2^ Elucidating the gene regulatory mechanisms underlying these tissues and organs is crucial for deciphering their development patterns and promoting clinical therapies. With rapid advances in omics sequencing technologies, we have begun to dissect how cells in various organs exert their specific functions at single-cell resolution^3^ and thus accumulate large amounts of single-cell data. However, gene expression is regulated at multiple levels, ranging from chromatin accessibility to post-transcriptional modification.^4,5^ This implies that comprehensively deciphering gene regulatory mechanisms solely through wet biological experiments is labor-intensive and time-consuming. The emergence of deep-learning models that capture and represent complex patterns in large datasets offers the opportunity to dissect multilevel and cross-species regulatory mechanisms.^6,7^

In recent years, foundation models such as BERT,^8^ GPT,^9^ PaLM,^10,11^ and LLaMA^12^ in natural language domains and DALL-E^13^ in visual domains have demonstrated remarkable performance in diverse downstream tasks. They typically adopt a paradigm involving initial pre-training on extensive data via self-supervised learning, followed by an adaptation step for specific downstream tasks via fine-tuning. Natural language serves as an abstract layer for understanding human activities. Similarly, the transcriptome can serve as a representative layer for understanding gene regulatory activities within biological systems. Several studies have utilized single-cell transcriptome data to construct pre-trained foundation models such as scGPT,^14^ Geneformer,^15^ UCE^16^ and scFoundation.^17^ These studies share the commonality of leveraging tens of millions of human single-cell transcriptomic profiles to pre-train foundation models and have demonstrated remarkable performance across a broad range of downstream tasks, such as cell clustering, cell type annotation, gene perturbation simulation, and drug target prediction.

Despite the extensive phenotypic diversity among vertebrates, gene regulatory networks exhibit a high level of conservation.^18,19^ Current models of gene regulation heavily rely on data from a single species. Thus, integrating datasets from different species represents a remarkable opportunity to unravel the intricate complexities of gene regulation.^2,20–22^ In the genomics era, a wealth of prior knowledge has been accumulated. This knowledge includes identification of crucial regulatory elements involved in gene expression, validated interactions between genes (gene regulatory network (GRN) and gene co-expression relationship), and definition of gene families with similar functional domains. This information significantly contributes to our comprehensive understanding of biological processes. Incorporating this knowledge into the pre-training process can substantially guide the model to learn universal gene regulatory mechanisms in a self-supervised manner.

In this study, we propose GeneCompass, a knowledge-informed cross-species foundation model pre-trained on scCompass-126M, which is the largest corpus encompassing over 120 million single-cell transcriptomes from humans and mice. After data pre-processing, 101,768,420 cells were utilized. The model incorporated prior biological knowledge, including promoter sequences, gene co-expression networks, gene family information, and transcription factor-target gene regulatory relationships. By fine-tuning our pre-trained model for various downstream tasks, GeneCompass achieved superior or comparable performance to state-of-the-art (SOTA) models across diverse biological contexts. Overall, our model represents a significant breakthrough in the development of foundational models for dissecting universal gene regulatory mechanisms from mouse to human and expediting the identification of crucial regulators of cell fate and potential targets for drug development.

## Results

### The architecture of GeneCompass and pre-training

**Fig. 1:**
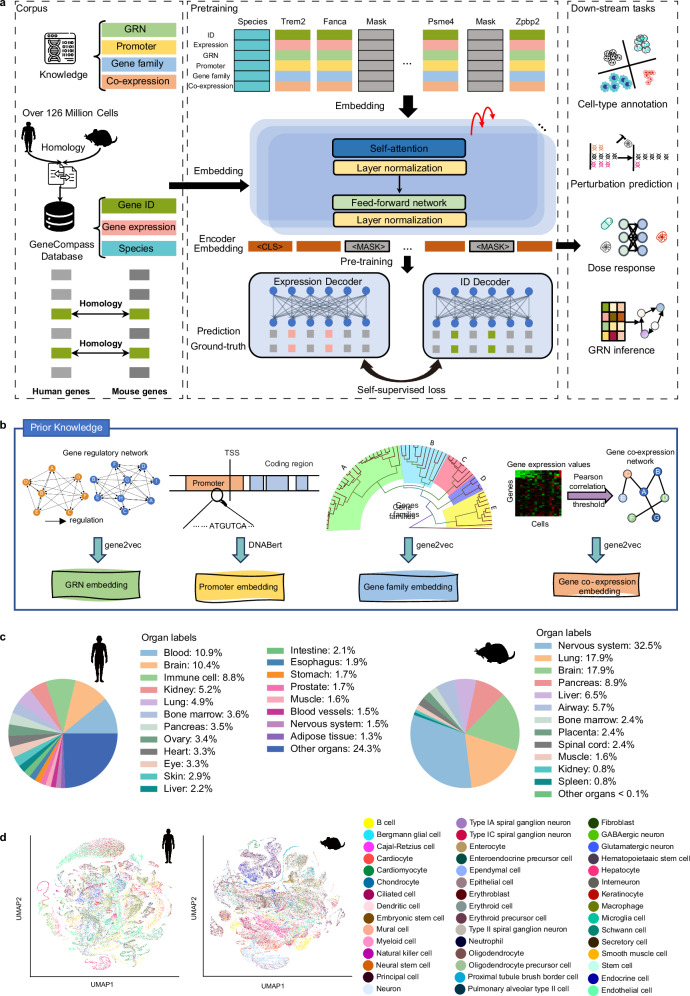
GeneCompass architecture and pre-training corpus. **a** The framework of GeneCompass. The model was pre-trained on large-scale single-cell transcriptomes of humans and mice and used for multiple downstream tasks, including cell-type annotation, perturbation prediction, dosage response prediction, GRN inference, and etc. **b** Embedding of four types of prior knowledge, including GRN, promoter sequence, gene family and co-expression. **c** Organ types of humans and mice in scCompass-126M. **d** Uniform Manifold Approximation and Projection of different cell types of a sampled subset from scCompass-126M.

GeneCompass is a knowledge-informed cross-species foundation model pre-trained on a transcriptomic corpus of over 120 million human and mouse cells (Fig. 1a). Four types of prior biological knowledge (GRN, promoter information, gene family annotation, and gene co-expression relationship) were integrated into self-supervised pre-training of GeneCompass (Fig. 1b). Utilizing the self-attention mechanism for explicit context encoding,^23^ GeneCompass could understand the essence of cells and the intricate relationships among genes based on the input transcriptomes. The pre-trained GeneCompass was designed to be efficiently applied to various downstream biological tasks by further fine-tuning limited task-specific data.

We initiated the development of GeneCompass by constructing a large-scale pre-training corpus, scCompass-126M. This corpus consists of 126 million human and mouse single-cell transcriptomes collected from publicly available datasets covering an extensive range of organs and cell types (Fig. 1c, d; Supplementary information, Fig. S1a–c). To ensure data quality, cells with outlier gene expression were filtered, and 101.76 million single-cell transcriptomes in scCompass-126M were retained. Additionally, we retained informative genes with sufficient variability or expression levels across the datasets to capture the biological heterogeneity and cell-type-specific signatures (Supplementary information, Fig. S1a). To integrate human and mouse cells, homologous genes between the two species were uniformly represented using human Ensembl IDs. Genes that did not have homologs were labeled with their respective species-specific Ensembl IDs. In this study, the token dictionary comprised 17,465 homologous genes out of 36,092 genes (Supplementary information, Fig. S1b).

The current large-scale transcriptomic pre-training models primarily utilize relative gene rankings^15^ or binned gene expression values^14^ as inputs, leading to inadequate representations of the transcriptome. To overcome this limitation, we first selected the top 2048 genes to construct the context for each cell after normalizing and ranking their gene expression, which was also used by Geneformer,^15^ then we concatenated absolute gene expression values and the corresponding gene IDs (Fig. 1a), towards stronger supervision constraints in self-supervised learning for GeneCompass. To further enhance the capability of our pre-trained model, four different types of biological prior knowledge, including GRN, promoter information, gene family annotation, and gene co-expression relationship, were encoded into a unified embedding space^24,25^ (Fig. 1b; Supplementary information, Fig. S1d–f). An extra token denoting the species (human or mouse) information was pre-pended to each cell (Fig. 1a) to fulfill cross-species pre-training. GeneCompass integrated gene ID, expression value, and prior knowledge as gene inputs and utilized a 12-layer transformer framework^23^ to encode cells. Inspired by self-supervised learning in the natural language processing domain, the masked language modeling strategy^8^ was employed to randomly mask 15% of gene inputs in each cell. GeneCompass built surrogate self-supervised tasks of recovering the gene IDs and expression values of masked gene inputs simultaneously, which enhanced its ability to capture the intricate gene relationships in a context-aware manner (see “Materials and Methods”).

### GeneCompass captures inherent gene features and relationships across species

**Fig. 2:**
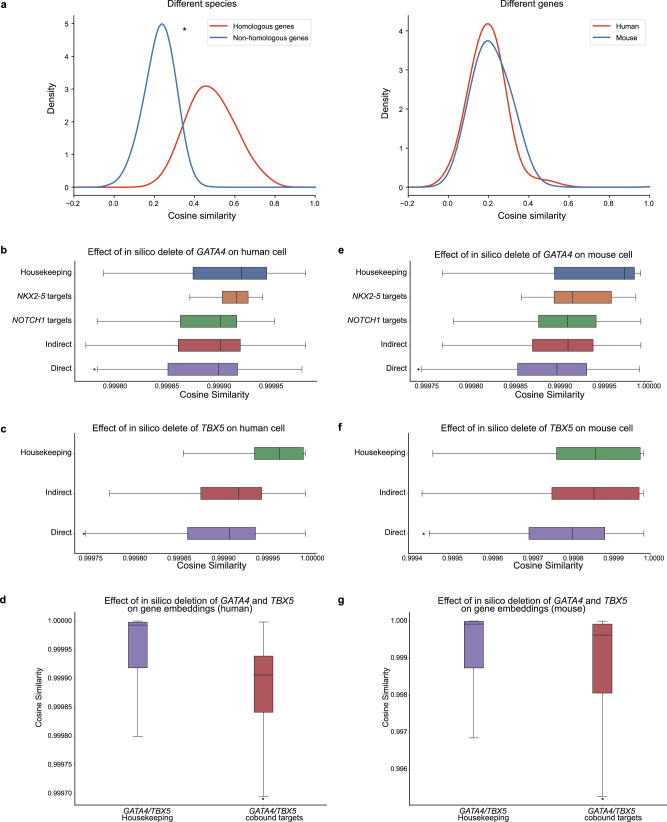
Analysis of gene embedding generated from GeneCompass. **a** Cosine similarity between homologous genes as well as non-homologous ones of different species (left panel), and that between different genes in the same mouse or human cell (right panel). **b**, **c** Effects of in silico deletion of *GATA4* and *TBX5* on different gene types, including their direct targets, indirect targets, *NOTCH1* targets, *NKX2-5* targets and housekeeping genes in human cardiomyocytes, respectively. **d** Effects of the individual and combined deletion of *GATA4* and *TBX5* as well as their combinatorial deletion with other genes that are not known to co-bind housekeeping genes and target genes in humans. **e**, **f** Effects of in silico deletion of *GATA4* and *TBX5* on different gene types, including their direct targets, indirect targets, *NOTCH1* targets, *NKX2-5* targets and housekeeping genes in mice which are obtained by homologous mapping. **g** Effects of the combined deletion of *GATA4* and *TBX5* on housekeeping genes and co-bound target genes in mice. (*P < 0.05, wilcoxon-test, NS no significance).

Homologous genes often retain similar expression patterns and functional roles, rendering known homology information an effective component for the integration of corpus across species.^26^ To validate whether the gene embeddings encoded by GeneCompass retained homology information, we randomly selected a total of 2000 B cells from human and mouse corpora, and compared the cosine similarity between the embeddings of homologous and non-homologous genes in different species. We found that the embeddings of homologous genes from GeneCompass were more similar than those of non-homologous genes, in terms of the statistical distribution of gene embedding similarity (Fig. 2a, left panel). The cosine similarity between different genes within the same mouse or human cell was also compared. The mean of the similarity distribution that was nearly zero showed the distinguishability between different gene embeddings of both human and mouse origin (Fig. 2a, right panel). Similar results were also observed in multiple cell types such as hepatocytes, macrophage cells, and a more generalized scenario by randomly selecting cells from different cell types, demonstrating that GeneCompass successfully captured the gene homology across species (Supplementary information, Fig. S2a–c). To test whether the similarity between homologs was derived from the prior knowledge or the self-supervised pre-training, we compared the cosine similarity of homologous genes in various scenarios, including pre-trained GeneCompass with prior knowledge, pre-trained GeneCompass without prior knowledge, unpre-trained GeneCompass with prior knowledge. The cosine similarity of non-homologous genes from the pre-trained GeneCompass with prior knowledge was also compared as a baseline (Supplementary information, Fig. S2d). The results showed that both the prior knowledge and the self-supervised pretraining contributed to the cross-species homology of GeneCompass, while the latter played a major role.

Next, we performed in silico gene deletion to validate whether GeneCompass could capture the gene regulatory relationships via pre-training. Previous reports have shown that *GATA4* and *TBX5* have a significant impact on congenital heart disease. The direct target genes that are known to be most aberrantly regulated by variants of *GATA4* and *TBX5* have been identified.^27,28^ We compared the cosine similarity of these genes after individual or simultaneous in silico deletion of *GATA4* and *TBX5* in human fetal cardiomyocytes. Consistent with the existing wet experimental results,^27,28^ in silico deletion of *GATA4* or *TBX5* individually had more impact on their direct target genes compared to their indirect target genes, housekeeping genes, and other congenital heart disease-related genes such as *NOTCH1* targets^29^ and *NKX2-5* targets^30^ (Fig. 2b, c). The difference between direct target genes and housekeeping genes was statistically significant by *t*-test. The in silico deletion of *GATA4* and *TBX5* simultaneously also suggested the cooperative impact on their co-bound target genes (defined by ChIP-Seq) and the impact on housekeeping genes was compared as a baseline (Fig. 2d). We also compared individual deletion and combinatorial deletion with other genes that they are not known to co-bind with them, and the results further proved that the pre-trained GeneCompass learned the co-bound effect of *GATA4* and *TBX5*. Considering the cross-species capability of GeneCompass, we conducted an array of the same experiments in mouse cardiomyocytes by homologously mapping *GATA4*, *TBX5*, and those corresponding targets and indirect genes from human to mouse, and obtained consistent experimental results (Fig. 2e–g; Supplementary information, Figs. S2e, S3e–h).

Additionally, we evaluated GeneCompass by in silico deleting more transcription factors (TFs) in various cell types of different species, i.e., *STAT1* on human PBMC cells, *SMARCA4* on human acute myeloid leukemia cells, *CBX8* on mouse embryonic stem cells, and *MTA2* on mouse colonic epithelium cells. In silico deletion of these TFs had more impact on their corresponding targets from the ChIP-seq dataset than on those housekeeping genes (Supplementary information, Fig. S3a–d).

To further validate the GeneCompass-learned gene regulatory relationships, we systematically evaluated GeneCompass’s ability in GRN recognition via two new experiments, an in silico gene deletion experiment and a gene perturbation simulation experiment. The results, illustrated in Supplementary information, Fig. S4a, showed that the gene regulation relationship obtained from the in silico perturbation suppressed the random guessing. Besides, we conducted the gene perturbation simulation using a former reported method from scGPT.^14^ The results on CREB1, BLHE40, and DDIT3 demonstrated that GeneCompass could preserve the gene–gene relationship for the perturbed conditions (Supplementary information, Fig. S4b, c).

Therefore, the extensive experiments demonstrated that the gene embeddings encoded by pre-trained GeneCompass could capture the inherent gene features and further learn gene regulatory mechanisms across species.

### GeneCompass boosts cell-type annotation from single species to cross species

**Fig. 3:**
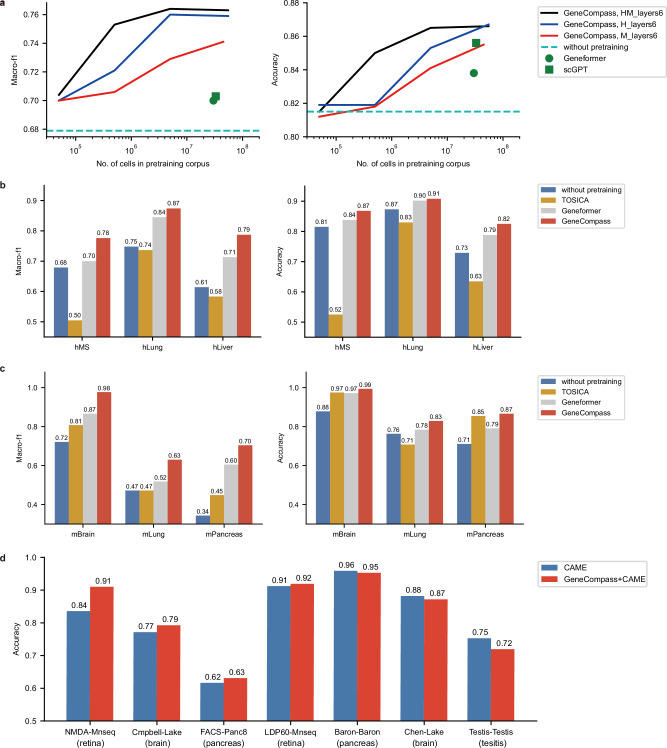
GeneCompass boosts the performance of cell-type annotations from single species to cross species. **a** Comparison of the performance of GeneCompass and other baseline methods on the downstream task of cell type annotation in the human multiple sclerosis (hMS) dataset. GeneCompass was pre-trained using human & mouse (HM, black line), human (H, blue line), and mouse (M, red line) single-cell transcriptome corpus with different cell numbers. The green circle point and green square point represent Geneformer and scGPT, respectively. “Layers6” denotes GeneCompass with a 6-layer transformer. **b** Comparison of the performance of GeneCompass and other baseline methods on hMS, hLung, and hLiver datasets. **c** Comparison of the performance of GeneCompass and other baseline methods on mBrain, mLung, and mPancreas datasets. **d** Comparison of the performance of GeneCompass+CAME with original CAME on cross-species cell type annotation (Mouse and human data were used as reference and target species, respectively). A 7.5% improvement was observed in NMDA-Mnseq, a retina dataset (the first column). The datasets in **b**–**d** derived from humans and mice are marked as “h” and “m”, respectively. Detailed information on datasets can be found in the Supplementary Methods. “Without pre-training” denotes that the parameters of GeneCompass were randomly initialized and fine-tuned directly without the pre-training process.

Although existing methods have shown decent performance in cell-type annotation, they only focus on single-species tasks. GeneCompass is pre-trained using the cross-species corpus and four types of prior knowledge, which could potentially boost cell-type annotation from single species to cross species. We observed that the performance of GeneCompass continuously improved as the pre-training corpus size increased for cell-type annotation on the human multiple sclerosis dataset (Supplementary Methods). With the same size of human pre-training corpus, GeneCompass (blue line) was consistently better than other benchmarked methods, i.e., Geneformer^15^ (green circle point) and scGPT (green square point), in terms of macro-f1 and accuracy, despite using a 6-layer transformer (Fig. 3a). Additionally, GeneCompass pre-trained with a combined human and mouse corpus (black line) demonstrated superior performance compared to models trained solely on human data or models trained on equivalent amounts of mouse data. These results indicated that incorporating another species’ data could enhance the performance of downstream tasks in one target species (Fig. 3a). Furthermore, we showed that GeneCompass with a 12-layer transformer performed better than that of the 6-layer transformer (Supplementary information, Fig. S5b). After that, we compared the performance of GeneCompass with or without this knowledge on the cell-type annotation task, the results of which demonstrated the advantage of infusing the prior information (Supplementary information, Fig. S5a and Table S2).

Next, to evaluate the capability of GeneCompass for single-species cell-type annotation tasks, we performed a comprehensive analysis of diverse organ datasets from humans and mice. A comprehensive comparison of four models, GeneCompass without pretraining, TOSICA,^31^ Geneformer,^15^ and pre-trained GeneCompass, was conducted in different human datasets, i.e., multiple sclerosis (hMS), lung (hLung) and liver (hLiver), and diverse mouse datasets, i.e., brain (mBrain), lung (mLung) and pancreas (mPancreas) (Supplementary Methods). We observed that pre-trained GeneCompass improved macro-f1 by 10%–18% than the case without pre-training, by 21%–28% than TOSICA, and by 3%–8% than Geneformer in human datasets (Fig. 3b). Meanwhile, pre-trained GeneCompass improved macro-f1 by 16%–36% than the case without pre-training, by 16%–25% than TOSICA, and by 10%–19% than Geneformer in mouse datasets (Fig. 3c). Fine-grained analysis showed that the pre-trained GeneCompass achieved higher recall on 16 of the 18 cell types in the mPancreas dataset comparing with TOSICA^31^ (Supplementary information, Fig. S5c, d). What’s more, compared with Geneformer, no matter the original one or the retrained one with the same corpus as GeneCompass, GeneCompass showed better performances on cell-type annotation (Supplementary information, Tables S3, S4). We could see that the performance improvement of GeneCompass was attributed to both pre-training input data and model architectures. These results indicated the superiority of GeneCompass on cell-type annotation tasks by pre-training a large-scale cross-species corpus.

To explore the capability of GeneCompass for cross-species cell-type annotation tasks, we integrated GeneCompass with SOTA method CAME.^17^ Gene embeddings generated by GeneCompass were utilized as the initial gene node features within CAME (Supplementary information, Fig. S5e). We utilized mouse cell type as a reference to annotate human cells on seven paired datasets from four distinct organs (retina, brain, pancreas, and testis). Following the integration of GeneCompass, we observed an overall comparable performance with an improvement in four of the seven paired datasets when comparing with CAME (Fig. 3d; Supplementary information, Fig. S5f). It is noteworthy that in a complex cross-species annotation task such as the retina (NMDA-Mnseq, the first column), which involves over 12 different cell types, we observed a 7.5% improvement compared to CAME, a leading specialized cross-species cell annotation tool. This improvement was achieved simply by replacing the gene information embeddings generated during the process with the embeddings generated by GeneCompass. This result highlights the potential of boosting cross-species tasks by GeneCompass.

In summary, the knowledge-informed cross-species GeneCompass pre-trained with over 120 million human and mouse cells outperformed the most advanced methods on single-species cell-type annotation tasks and exhibited great potential on cross-species tasks.

### Pre-trained gene embeddings improve the prediction performance in multiple biological contexts

**Fig. 4:**
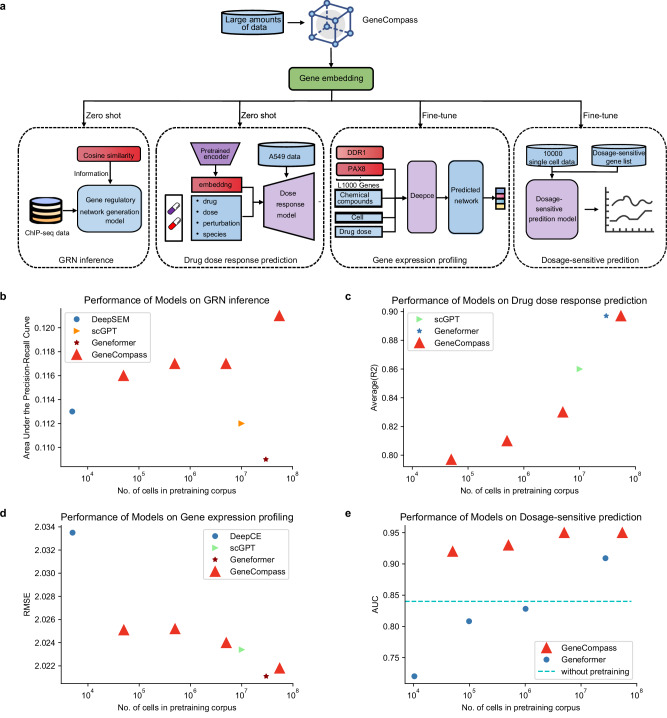
GeneCompass demonstrates enhanced performance for GRN inference, drug dose response prediction, gene expression profile prediction, and gene dosage sensitivity prediction tasks. **a** The workflow of integrating gene embeddings generated from GeneCompass to four downstream tasks: GRN inference, drug dose response prediction, gene expression profiling and gene dosage sensitivity prediction. **b** Performance comparison of each model on the GRN inference task in terms of AUPRC. The red line denotes the results of GeneCompass trained by different amounts of data. The blue, orange and brown dots represent results of DeepSEM, scGPT and Geneformer, respectively. **c** Performance comparison, in terms of R-squared value, for each model is conducted on the drug dose response prediction task. The red line denotes the results of GeneCompass trained by different amounts of data. The green and blue dots represent results of scGPT and Geneformer, respectively. **d** Performance comparison of each model on the gene expression profile prediction task. Root Mean Squared Error is applied as the metric. The red line denotes the results of GeneCompass trained by different amounts of data. The blue, green and brown dots represent results of DeepCE, scGPT and Geneformer, respectively. **e** Performance comparison of each model on the dosage sensitivity prediction task. We use AUC as the metric. The red and blue lines denote the results of GeneCompass and Geneformer, respectively, trained by different amounts of data. The dashed line represents the result of GeneCompass without pretraining.

To further investigate the capabilities of the gene embeddings encoded in GeneCompass, we applied them to several downstream prediction tasks, including GRN inference, drug dose-response prediction, gene expression profiling, and gene dosage sensitivity (Fig. 4a; Supplementary information, Fig. S6).

GRN provides information on gene regulation and signal transduction, offering insights into gene expression patterns and key regulatory genes in diseases. During the pretraining process, GeneCompass effectively captured gene regulatory relationships within its gene embeddings, which could potentially enhance the GRN inference application. Thus, we generated gene–gene relationship information based on the cosine similarity between the gene embeddings from GeneCompass by turning the pairwise similarities into a binary adjacency matrix with an optimized universal threshold at 0.4. This information was then used to update the output GRNs from DeepSEM,^32^ an advanced GRN inference tool. Moreover, we conducted a similar assay using gene embeddings generated by other pre-trained models, namely Geneformer and scGPT, respectively. The GRN inference task was known to be a typical imbalanced problem where only a small percentage of gene pairs had a regulatory relationship. Thus, we used the Area Under the Precision-Recall Curve (AUPRC) as the evaluation metric for assessing the performance, which was also derived from DeepSEM.^32^ The ground truth dataset was based on ChIP-Seq and de-duplicated against the prior knowledge of PECA GRN. Then through this metric, we observed that GeneCompass achieved the best GRN inference performance compared to scGPT, Geneformer, and vanilla DeepSEM (Fig. 4b; Supplementary information, Fig. S7a–c).

Predicting drug responses to gene expression changes to different types and dosages of drugs is crucial for drug functional evaluation. To validate the potential benefits of gene embeddings generated by GeneCompass and other pre-trained models for this task, we incorporated them into the Compositional Perturbation Autoencoder (CPA)^33^ framework to predict the expression changes of specific genes (as an example, *MDM2*). GeneCompass exhibited constantly better performance with increasingly enlarged data volume. GeneCompass achieved the highest scores, the same as Geneformer (Fig. 4c; Supplementary information, Fig. S7d), and demonstrated lower variance across different drug conditions compared to the other models (Supplementary information, Figs. S6d, S7). In addition to predicting single gene changes, we also incorporated gene embeddings generated by GeneCompass into DeepCE,^34^ a widely used model for predicting drug-induced changes in gene expression profiles, to assess its impact on model performance. As the data volume increased, the performance of GeneCompass was consistently improved. The performance of the final GeneCompass was comparable with that of Geneformer and better than other pre-trained models (Fig. 4d).

Determining dosage-sensitive genes is of vital importance for interpreting copy number variations (CNVs) in genetic diagnosis. Here, we fine-tuned GeneCompass to identify the dosage-sensitive genes on predefined dosage-sensitive and non-sensitive gene datasets.^15^ We observed that as the number of cells used for pre-training increased, the predictive performance of GeneCompass in terms of area under the receiver operating characteristic curve (AUC) consistently improved, reaching 0.95 (Fig. 4e). Utilizing an identical amount of training dataset, GeneCompass achieved superior performance compared to Geneformer. This improved efficacy could be attributed to the strategic inclusion of prior knowledges during pretraining.

In summary, GeneCompass demonstrated promising results in multiple tasks, including GRN inference, drug dose response prediction, gene expression profiling, and gene dosage sensitivity prediction tasks. The performance of GeneCompass proved its adaptability and effectiveness in a variety of downstream biological tasks. What is more, the extensive experiments also proved the plentful corpus and novel architecture improved the performance of GeneCompass on the full-panel of downstream tasks (Supplementary information, Tables S3, S4).

### Pre-trained gene embedding improves gene perturbation prediction

**Fig. 5:**
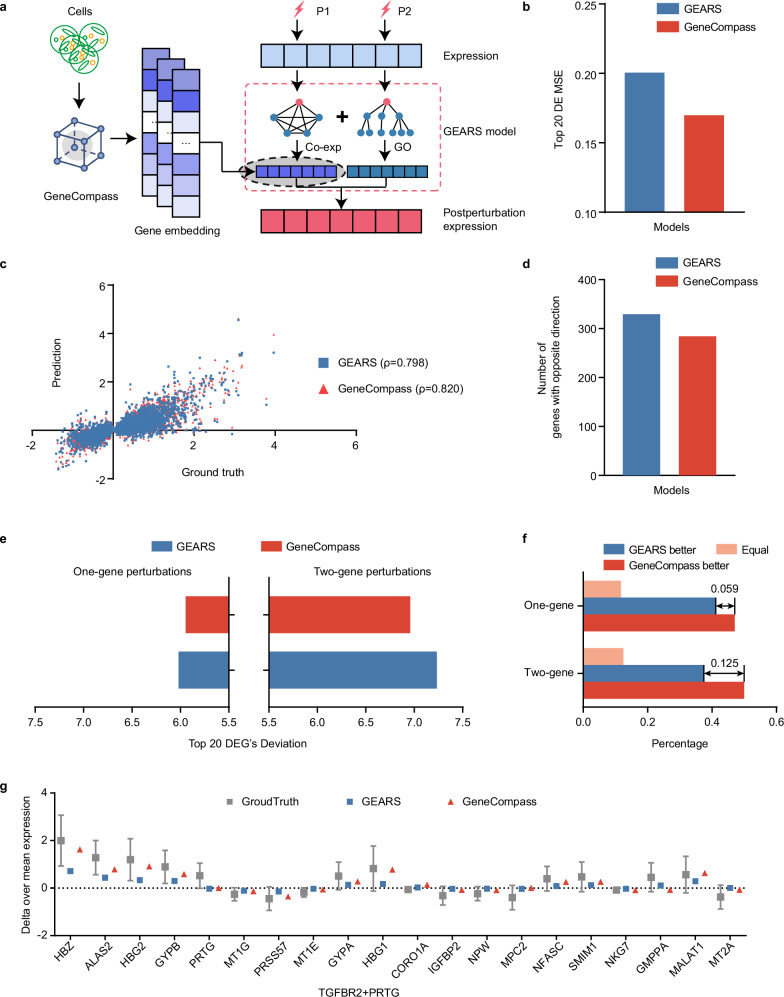
GeneCompass shows enhanced performance for the gene perturbation prediction task. **a** The workflow of GeneCompass for the perturbation prediction task. **b** MSE in predicting the expression changes in the top 20 DEGs by GeneCompass and GEARS. MSE only considered on the top 20 most DEGs. **c** Scatter plot of the predicted and true changes in gene expression. Each dot represents a specific gene, and Spearman’s correlation is marked as “$\rho$”. **d** Total number of the top 20 DEGs genes where the predicted post-perturbation differential expression was in the incorrect direction of the ground truth. **e** Expression deviation between the predicted and true changes in gene expression for the top 20 DEGs analyzed by GeneCompass and GEARS. **f** Percentage of perturbations that exhibited a smaller deviation between the prediction results and ground truth when comparing GeneCompass with GEARs, using the deviation in the top 20 DEGs as the criterion. “GeneCompass better” is defined as GeneCompass having a smaller deviation than GEARS. **g** Expression changes for the combined *TGFBR2* and *PRTG* perturbation in true experiment post perturbation were predicted by GeneCompass and GEARS. The grey error bar denotes the ground truth of mean gene expression change with standard deviation after perturbing the gene combination *TGFBR2* and *PRTG* (*n* = 205). The red triangle symbol shows the gene expression change predicted by GeneCompass with *TGFBR2* and *PRTG* perturbation excluded during training. The blue square symbol shows the gene expression change predicted by GEARS.

Although gene dosage has a significant impact on disease and drug treatment, functional mutations directly affect gene function and lead to a wide range of cellular changes. We attempted to leverage the gene embedding provided by GeneCompass to predict global gene expression changes resulting from perturbations caused by functional gene mutations. We integrated GeneCompass gene embedding into the advanced perturbation prediction tool GEARS^35^ by replacing the original gene embedding that was learned from the co-expression knowledge graph in the original procedure (Fig. 5a). This led to a decrease of 15.4% in the mean squared error (MSE) for the top 20 differentially expressed genes (DEGs) when training on Norman perturb-seq dataset,^36^ indicating a lower discrepancy between the predicted and actual expression changes for these critical genes (Fig. 5b).

We then ran a hold-out test to evaluate the GeneCompass performance for 102 one-gene and 128 two-gene perturbation predictions. The Spearman’s rho between the predicted and true changes in gene expression after perturbations showed that GeneCompass had an improvement of 2.2% compared to GEARS, increasing from 79.8% to 82.0% (Fig. 5c). This enhancement was observed in both one-gene and two-gene perturbations (Supplementary information, Fig. S9a–e). Next, we investigated whether GeneCompass could more accurately predict the correct direction of changes in gene expression after perturbations. The top 20 DEGs with incorrect directions for each perturbation prediction were summarized. Comparing the results with those predicted by GEARS, we observed a decrease of 13.7% from 336 to 290 DEGs with incorrect directions (Fig. 5d; Supplementary information, Fig. S9f).

Furthermore, the deviation analysis for each perturbation prediction indicated that GeneCompass exhibited lower deviations against the ground truth in the top 20 DEGs for both one-gene and two-gene perturbations, as compared to GEARS (Fig. 5e; Supplementary information, Fig. S9g). GeneCompass provided a deviation reduction of 5.9% for one-gene perturbations and 12.5% for two-gene perturbations compared with GEARS (Fig. 5f). As an example, we showed that the prediction results for 17/20 DEGs from GeneCompass were more accurate than those from GEARS when perturbing *TGFBR2* and *PTRG* genes (Fig. 5g). Similar results could also be observed in other gene perturbations (Supplementary information, Fig. S9h–j). In summary, gene embedding in GeneCompass provided a more effective representation of relationships between genes, enhancing the prediction of gene perturbations.

### GeneCompass enables cell fate prediction and identifies key regulatory factors

**Fig. 6:**
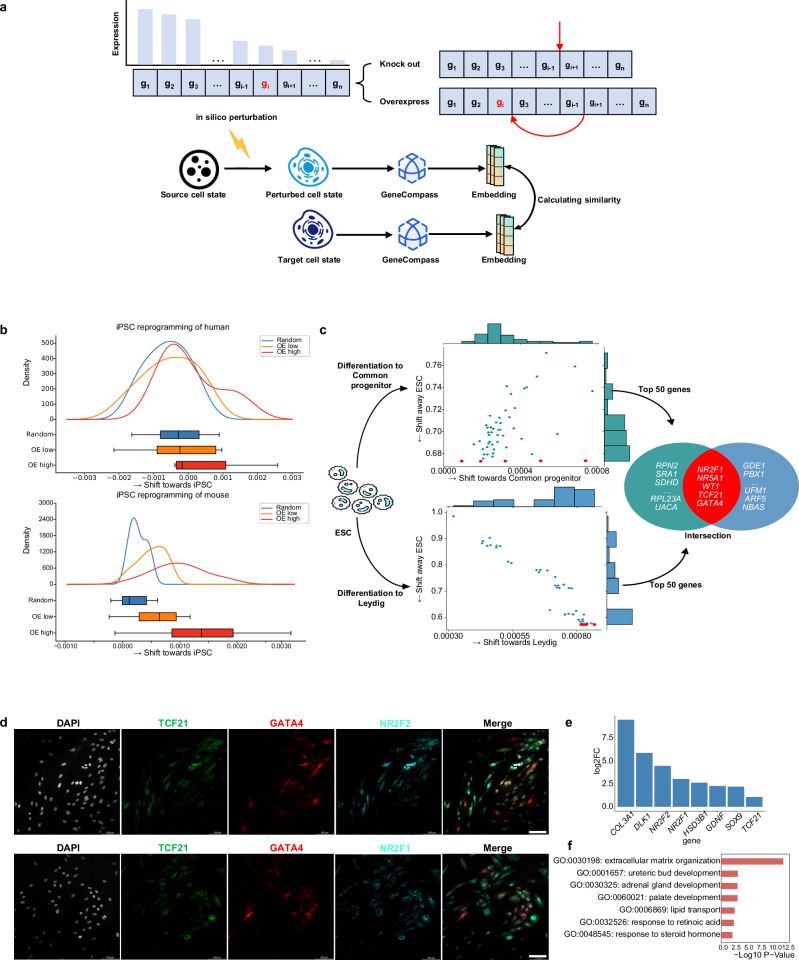
In silico quantitative perturbation for cell reprogramming and differentiation. **a** Diagram of in silico cell fate transition. In silico knockout or overexpression experiment is performed by removing or shifting the highlighted gene in red forward within the ranking genes. **b** In silico low-level or high-level overexpression of OSKM is performed in human (upper) or mouse (bottom) fibroblasts to calculate the cosine similarity of the simulated cell states with iPSCs. In silico overexpression of four other random genes is used as control. In each simulation group, all embedding pairs between perturbed fibroblast cells and iPSCs are used to compute the cosine similarity. The cosine similarity of all pairs in each group is simultaneously presented using probability density and box plots. **c** Distribution of candidate genes that drive the shift of cell embeddings towards Leydig cell status and gonadal progenitor status in response to in silico overexpression in human ESC cells. Top 50 genes shifting towards Leydig cell (lower) or gonadal progenitor (upper) status and away from the ESC status are presented. Five genes in the intersection set of Venn diagram are selected as candidate genes for gonadal differentiation. **d** Protein co-immunofluorescence staining for markers of interstitial/Leydig lineage and Sertoli cells with GATA4 (GATA4^+^, red; TCF21^+^, green; NR2F2/NR2F1^+^, cyan),). Scale bars: 100${{\rm{\mu }}}{{\rm{m}}}$ **e** The identification of upregulated gonadal lineage-related marker genes in the GATA4 overexpression group compared to cells derived from wild-type ESCs, with fold changes exceeding 2-fold. **f** Gene ontology (GO) enrichment analysis was performed using DAVID for the total up-regulated genes with a 2-fold change in the GATA4 overexpression group compared to cells derived from wild-type ESCs. (*P < 0.05, Wilcoxon-test).

Owing to that both the absolute expression value and relative ranking indices of genes were masked and reconstructed during the self-supervised pre-training, GeneCompass could capture intricate regulatory mechanisms, thus enabling in silico quantitative gene perturbation (Fig. 6a). To verify this capacity, we simulated iPSCs induction procedure, similar to the existing work^15^ (Supplementary information, Fig. S10b), a well-characterized reprogramming paradigm, via in silico overexpression of OSKM genes^37^ (*OCT4*, *SOX2*, *KLF4* and *c-MYC*) in human fibroblasts. We set two levels of overexpression for OSKM genes: the median value of the gene across the corpus (low-level overexpression) and the maximum value in the cell (high-level overexpression). Compared to the control group that overexpressed four other random genes, cells with both levels of in silico OSKM overexpression exhibited a shift towards the iPSC state. Notably, cells with a high-level overexpression of OSKM were shifted further towards the iPSC state than those with a low-level overexpression of OSKM, reflecting a precise simulation of cell reprogramming. Consistent results were observed in mouse fibroblasts (Fig. 6b). Then GeneCompass was evaluated on in silico quantitative knockout tasks in cell differentiation processes. *Zbtb11* and *Zfp131* are reported to be essential TFs for the maintenance of pluripotency in mouse embryonic stem cells (ESCs).^38^ Deletion of *Zbtb11* and *Zfp131* could induce endoderm differentiation. In our study, in silico quantitative knockouts of *Zfp131* and *Zbtb11* were performed in mouse ESCs by progressively reducing their expression levels to half, quarter, and zero, respectively. Consistent with the actual knockout results,^38^ we observed that all simulated knockout cells exhibited a shift towards the endoderm state. Importantly, we found a positive correlation between the extent of shifting and the in silico knockout levels (Supplementary information, Fig. S10a).

Next, we utilized GeneCompass to predict key regulatory factors in cell fate transitions through in silico analysis, with the aim of promoting efficiency of wet experiments and uncovering novel mechanisms. Here, we conducted an experiment on the differentiation of human ESCs to the gonadal lineage cells (Fig. 6c; Supplementary information, Fig. S10c). Specifically, each gene in the initial ESCs was in silico overexpressed to generate a cell embedding that represented the simulated differentiation state. By comparing the cosine similarities among initial, simulated, and target cell embeddings, we identified the top five genes, i.e., *NR2F1, NR5A1, WT1, TCF21*, and *GATA4*, whose simulated cell embeddings exhibited higher similarity to both gonadal progenitors and mature Leydig cells while lower similarity to the original ESCs (Fig. 6c, see “Materials and Methods”). Interestingly, all five of these genes are transcription factors, and three of them, i.e., *WT1*, *NR5A1*, and *NR2F1*, have been reported to be essential for mouse gonadal development in vivo.^39–45^ Hence, these genes could be key factors that trigger the differentiation of ESCs into gonadal progenitor cells.

To further validate the role of these genes, we individually overexpressed *NR5A1* and *GATA4* in ESCs and subsequently induced differentiation towards the gonadal lineage (Supplementary information, Fig. S10d). Immunofluorescence results demonstrated that overexpression of either gene alone in human ESCs could induce gonadal gene programs, as evidenced by the activation of marker genes associated with the gonadal progenitors (Fig. 6d; Supplementary information, Fig. S10e). Additionally, a comprehensive transcriptome comparison analysis revealed a significant upregulation of known marker genes associated with reproductive development in *NR5A1-* or *GATA4-*overexpressed cells, compared to ESCs (Fig. 6e; Supplementary information, Fig. S10f). The DEGs were found to be enriched in gonadal-related biological processes (Fig. 6f; Supplementary information, Fig. S10g). Intuitively, *NR5A1-* and *GATA4-*overexpressing cells directly upregulated the *RBP1* gene involved in maintaining retinoic acid homeostasis and the related genes *STAR* and *HSD3β1* involved in steroid hormone synthesis in testicular cells (Fig. S10h, i). These results suggested that the predicted genes indeed played an important role in the gonadal differentiation from human ESCs.

In summary, GeneCompass exhibited the capability of cell fate prediction and key regulatory factor identification which could be applied to refine the efficiency of wet experiments and reveal novel mechanisms.

## Discussion

In this study, we introduced GeneCompass, a large-scale pre-trained model that integrates 126 million cross-species single-cell transcriptomes with four types of prior known biological knowledge. GeneCompass used a deep learning architecture based on Transformer’s self-attention mechanism to capture long-range dynamic associations between different genes in different cellular contexts. During pre-training, genes were ranked and combined with expression values for dual encoding input. This enables GeneCompass to effectively and sensitively extract relationships between genes and provide a more precise analysis of gene–gene interactions under specific conditions.

We found that GeneCompass using large-scale cross-species data for downstream tasks of single species followed the scaling law: larger-scale multi-species pre-training data produce superior pre-training representations compared to single-species data, further enhancing downstream tasks performance. This finding further confirms the existence of conserved gene regulatory patterns across species, which can be learned and understood by pre-training models. It also suggests that as species and data expand, model performance is expected to be continuously improved.

GeneCompass represents a knowledge-embedded cross-species pre-trained large foundation model in life sciences, enabling transfer learning for multiple cross-species downstream tasks. Compared to the existing models, it achieved better performance in various downstream tasks such as cell type annotation, GRN inference, drug dose response prediction, gene expression profiling, and gene dosage sensitivity prediction as well as quantitative gene perturbation predictions. These results proved the effectiveness of the strategy that pre-training a foundation model on multi-species large-scale unlabeled data and subsequent fine-tuning on limited task-specific data could make it a promising universal solution to various important biological problems related to gene–cell features. In addition, we conducted extensive cross-validation experiments to investigate the impact of incorporating various prior knowledge on pre-training performance by utilizing full panel of downstream tasks. These results showed that infusion of prior knowledge could promote the understanding of complex feature associations between biological data by the pre-trained foundation model.

However, there still is potential for improvement. Our model incorporated information from only two species: human and mouse. When attempting to include data from other species, we suspected that species-specific gene expression patterns may offset the benefits of enlarged data size. In addition to current prior knowledge, other essential information such as enhancers and protein sequences should also be explored. Furthermore, apart from transcriptional data at the single-cell level, a wealth of epigenomic, proteomic, and metabolomic data would provide richer insights into gene regulation. Investigating effective strategies for integrating multimodal information into models is a pivotal avenue for future research.

GeneCompass demonstrates promising performance for multiple downstream tasks. With ongoing evolution and increased adoption, it holds the promise to provide substantial value in optimizing cell-fate prediction and uncovering key regulatory factors. This can open up new avenues for its clinical application, for example, disease target gene discovery, tumor drug screening, and drug toxicity prediction. In the future, we anticipate that the fusion of large foundation models and wet experiments will create a novel paradigm in life science research, catalyzing advancements in various fields.

## Materials and methods

### Collecting and preprocessing of multi-species training data

We constructed a large-scale pre-training corpus, scCompass-126M, comprising more than 120 million single-cell transcriptomes derived from humans and mice. In detail, we obtained 53,568,337 human single cells, 48,200,083 mouse single cells, and a total of 101,768,420 human and mouse single cells. More than 90% (about 94.41%) of the data were obtained from publicly available datasets from various sources, including the Gene Expression Omnibus of the National Center for Biotechnology Information (NCBI), European Molecular Biology Laboratory-European Bioinformatics Institute (EMBL-EBI) ArrayExpress, and China National Center for Bioinformation (CNCB) (Supplementary information, Table S1). Raw sequence data were downloaded from these databases, with different regions in the world, each sample file was labeled with a unique ID, and the gene count matrices were obtained using Cell Ranger.^46^ The other part of the data in the gene count matrix were mainly downloaded from CELLxGENE database.

A unified bioinformatics processing pipeline was utilized for high-quality data filtering (Supplementary information, Fig. S1a). The pipeline consisted of the following steps: filtering cells with less than 200 expressed genes, filtering samples with less than 4 cells, filtering cells with more than 7 expressed protein-coding or miRNA genes, filtering cells with a proportion of mitochondrial genes exceeding 15%, filtering cells with an expressed gene number exceeding three standard deviations from the mean number among all cells in the gene expression matrix for each sample, and dropping genes not in the core gene list. Informative gene annotations from Ensembl were used to define a core gene list including protein-coding genes, lncRNAs, and miRNAs. Categories like pseudogenes, tRNAs, rRNAs, and other non-capturable loci by current single-cell transcriptomics were excluded from the analysis.

Our pretraining corpus included disease cells, cancer cells, and immortalized cell lines. Metadata descriptions in the datasets, including single-cell perturbation, cancer cell identification, gender distribution, and cell differentiation time, were used to assess the diversity of the datasets (Supplementary information, Fig. S1c).

### GeneCompass architecture and pre-training

#### GeneCompass architecture

GeneCompass employed self-attention transformer to encode each single-cell transcriptome data. We denoted the number of transformer layers as *L*, the number of self-attention heads as *H*, and the hidden size as *D*. We primarily utilized a transformer with $L=12,{H}=12,{D}=768$, whose total parameters reached over 100,000,000. GeneCompass operated on a sequence of 2048 genes for each cell sample, with each sequence obtained according to the corresponding ranked high expression. Given a gene, the information including gene ID, expression value, corresponding prior knowledge (promoter, GRN, gene family, and co-expression), and a special token indicating species were concatenated and further encoded into 768-dimension embeddings. A position embedding indicating the gene ranking was also added to the input. Gaussian Error Linear Units (GELUs) were employed for nonlinear activation, and the dropout probability for both self-attention and dense layers is 0.02 (standard deviation of the initializer for weight matrices is 0.02; epsilon for layer-normalization layers is 1 × 10^–12^). Code for model configuration, data loading, and training was implemented by Pytorch and Huggingface Transformers library.^47^ Furthermore, we extended the library for inputting scalable external knowledge.

#### GeneCompass pre-training and optimization

Inspired by self-supervised learning in the NLP domain, a masked language modeling strategy^8^ was employed to randomly mask genes including their IDs, expressions, and prior knowledge during the pre-training. To be detailed, 15% of the genes were selected randomly to be masked in each cell. Compared with existing studies,^14,15,17^ GeneCompass built a multi-task learning paradigm to predict both expression value and ID of the masked genes based on the encoded gene embedding in the meanwhile. We used the MSE loss for gene expression prediction, which was defined as follows:

$${{{\mathscr{L}}}}_{\exp }=\frac{1}{{n}_{{\mbox{m}}}}{\sum }_{j=1}^{{n}_{{\mbox{m}}}}{\left({\widetilde{x}}_{j}^{(i)}-{x}_{j}^{(j)}\right)}^{2}$$

where ${n}_{{\mbox{m}}}$ denoted the number of masked genes, ${\widetilde{x}}_{j}^{(i)}$ denoted the predicted gene expression and the ground truth expression was denoted as ${x}_{j}^{(i)}$. Cross-entropy (CE) loss was employed for gene ID prediction, which was defined as follows:

$${{{\mathscr{L}}}}_{{ID}}=-\frac{1}{{n}_{{\mbox{m}}}}{\sum }_{j=1}^{{n}_{{\mbox{m}}}}p\left({x}_{j}\right){{\mathrm{log}}}\, q({x}_{j})$$

where $p(x)$ denoted the probability of the real gene ID, and $q(x)$ denoted the probability of predicted ID. The overall training objective combines the MSE loss for gene expression and CE loss for gene ID:

$$\min (1-\beta ){{{\mathscr{L}}}}_{{ID}}+\beta {{{\mathscr{L}}}}_{{ID}}$$

where *β* is the hyperparameter to balance the tasks’ gradient.

The pre-training parameters were as follows: to make full use of GPU, the batch size is adjusted to the maximum allowable value of 10 for the 12-layer transformer model. The learning rate was set to linear decay with 10,000 warm-up steps, and the maximum learning rate was 5e–5 using the AdamW optimizer. GeneCompass was pre-trained for 3 epochs, in which case the loss basically did not decrease. The whole pre-training process was accomplished in 9 days using 4$\times$8 NVIDIA A800 GPUs.

#### Cell and gene embedding

We could obtain the cell and gene embeddings from the output of last layer in GeneCompass. GeneCompass encoded each gene into a 768-dimension embedding, which contained its context information in the cell. And the embedding of the special token was used as the cell embedding that indicated the cell status.

#### Ablation experiments

An ablation study of GeneCompass with the inclusion of different kinds of prior knowledge is performed using the full human single-cells corpus (~55 millions). To avoid consuming too much computing power, GeneCompass utilized a smaller self-attention transformer with $L=6,{H}=4,{D}=256$. Specifically, GeneCompass with inclusion of zero, one, and all kinds of prior knowledges, respectively, were used for pre-training. To compare with Geneformer, we also re-trained Geneformer using the same dataset as GeneCompass. To clarify the impact of input data on model performance, we further built two small datasets of 5 million single cells by randomly selecting from Geneformer and GeneCompass corpus. Then, Geneformer and GeneCompass were pre-trained based on the corresponding dataset. The experimental results were based on fine-tuning these pre-trained models on full-panel multiple downstream tasks by cross-validation.

#### Downstream task fine-tuning

For a downstream task, the pretrained GeneCompass was further fully fine-tuned using limited data. A task-specific decoder (e.g., a dense layer) was appended to the 12-layer transformer encoder of GeneCompass. For those comparative methods of downstream tasks, all the hyperparmeters were fine-tuned with a same process as GeneCompass to guarantee a fair comparison. Specifically, we firstly used the official codebases of the comparative methods for downstream tasks; then, we performed the same parameter fine-tuning process on the models (such as learning rate, batch size, and number of iterations); finally, we used the best performance of each model achieved to compare.

### Knowledge embedding and incorporation

Four types of prior biological knowledge of a gene were encoded into the same 768-dimension of embedding and incorporated with gene ID and expression value, which included GRN, promoter information, gene family annotation and gene co-expression relationship.

#### GRN embeddings

We used paired gene expression and chromatin accessibility data from the Encyclopedia of DNA Elements (ENCODE) to construct the PECA2^48^ GRN. In total, 84 mouse and 76 human GRNs were generated (Supplementary information, Fig. S1f). We then embedded these gene pairs with regulatory relationships into vectors using the gene2vec method.^25^

#### Promoter embeddings

The promoter is a noncoding sequence of a gene that serves as an activating signal for gene transcription. The promoter for each gene consists of 2500 bases, including upstream 500 bases before the transcription start site (TSS) and downstream 2000 bases. The promoter sequences were fine-tuned on the pre-trained model DNABert^24^ for 40 epochs to obtain promoter embeddings with 768 dimensions.

#### Gene family embeddings

The human gene family data are from the HUGO Gene Nomenclature Committee (HGNC) database, and can be downloaded from https://www.genenames.org/download/custom/. The mouse gene family data were derived from the human gene family data based on the homologous genes between humans and mice, and the homologous genes are from the BioMart database, which is a sub-database of the Ensembl database. We analyzed the genes of two organisms and their family relationships as follows: 1539 gene families for mice and 1645 gene families for humans. Any two genes belonging to the same gene family were regarded as a gene pair to construct a gene pair list. All gene pairs were encoded by gene2vec method.^25^ We retained the structure of the initial gene2vec but modified the embedding dimension from 256 to 768 during training. Eventually, each gene was represented as a 768-dimension embedding (Supplementary information, Fig. S1e).

#### Co-expression embeddings

We employed a uniform sampling strategy by selecting 3000 cells from each gene expression matrix. This step aimed to comprehensively cover the range of gene expression, capture overall features, and avoid bias towards specific cell types or expression levels. We calculated the Pearson correlation coefficient (PCC) between two expressed genes (count ≥ 1) (Supplementary information, Fig. S1d). The nonzero-based correlation coefficient calculation avoided unnecessary calculations for zero values, ensuring the practicality and interpretability of the analysis results. Gene pairs with PCCs larger than 0.8 were selected to be embedded using the gene2vec method.

### Multiple downstream tasks

#### Gene embedding analysis

This task was designed to analyze the gene–gene interaction by performing in silico deletion without the comparison with perturbed conditions. The in silico perturbation was conducted by removing the target gene from single-cell transcriptome. The shift of each gene was calculated by comparing the similarity of gene embeddings between the origin and in silico perturbed status. The larger shift denoted the higher correlation with the perturbed gene. The GRN inference demonstrated in Supplementary information, Fig. S4a was performed based on in silico deletion of TFs in CHIP-Atlas related to PBMC cells on GSE43036. For each transcription factor, we calculated the precision of predicted TFs using GeneCompass and random selection.

#### Single-species cell type annotation

To simulate the application scenario of GeneCompass more realistically, we selected samples with different biological backgrounds and batches as the training set and testing set, respectively. To predict single-species cell types, a fully connected layer was added to the cell embeddings generated from GeneCompass. The cross-entropy loss was used as objective function. For human-specific and mouse-specific tasks, we compared pre-trained GeneCompass with GeneCompass without pre-training, Geneformer and TOSICA on human multiple sclerosis (hMS), lung (hLung) and liver (hLiver) datasets, and mouse brain (mBrain), lung (mLung) and pancreas (mPancreas) datasets. Details of the datasets used for model training and validation can be found in the Supplementary Methods. Through the hyperparameter tuning process, different models can obtain hyperparameters that were more suitable for the datasets, which also facilitates comparison between different methods. For GeneCompass, learning rate was set to 5e–5, batch size was set to 16, and number of training epochs is set to 50. For Geneformer, learning rate was set to 5e–5, batch size was set to 16, and number of training epochs was set to 50. For TOSICA, learning rate was set to 5e–5, batch size was set to 16, and number of training epochs was set to 20. For scGPT, we used the results from the corresponding dataset provided in the original article.

#### Cross-species cell type annotation

To predict cell types across species, we combined GeneCompass with CAME^49^ to obtain a heterogeneous graph neural network called GeneCompass+CAME, in which cells and genes were modeled as heterogeneous nodes (Supplementary information, Fig. S5e). Furthermore, similar to CAME, we created a heterogeneous graph with six heterogeneous types: “cell to gene”, “gene to cell”, “cell to cell”, “gene to gene”, “cell self-loop”, and “gene self-loop”, where we denoted the corresponding weights (shared across species) as ${W}_{{cg}}$, ${W}_{{gc}}$, ${W}_{{cc}}$, ${W}_{{gg}}$, ${W}_{c}$ and ${W}_{g}$, respectively. Unlike CAME, GeneCompass+CAME adopted gene embeddings from pre-trained GeneCompass as input.

Here, we denoted a gene expression matrix with N cells and M genes as $X\in {R}^{N\times M}$, and the corresponding pre-trained gene embeddings as ${X}_{g}\in {R}^{{M}^{{\prime} }\times P}$. The initial embedding (the 0-th layer) for each cell *i* was calculated as follows:

$${h}_{{c}_{i}}^{\left(0\right)}=\sigma \left({W}_{c}^{\left(0\right)}{{{x}_{e}}_{c}}_{i}+{b}_{c}^{\left(0\right)}\right),$$

where *σ* was the leaky ReLU activation function with a negative slope of 0.05, ${{{x}_{e}}_{c}}_{i}$ represented the embeddings generated from the selected homologous genes for cell *i*, ${W}_{c}^{\left(0\right)}$ and ${b}_{c}^{\left(0\right)}$ were learnable weight and bias vectors. In GeneCompass+CAME, we used the pretrained gene embedding ${h}_{{g}_{j}}^{\left(0\right)}$ to initialize each gene. We then aggregated the features of the corresponding neighboring cells to update the gene embedding:

$${h}_{{g}_{j}}^{\left(1\right)}=\sigma \left({h}_{{g}_{j}}^{\left(0\right)}+{\sum}_{i\in {{{\mathscr{N}}}}_{{g}_{j}}^{c}}\frac{1}{{z}_{{g}_{j},c}}{W}_{{cg}}^{\left(0\right)}{{{x}_{e}}_{c}}_{i}+{b}_{g}^{\left(0\right)}\right),$$

where ${{{\mathscr{N}}}}_{{g}_{j}}^{c}$ was the set of cells that express gene $j$, and ${W}_{{cg}}^{\left(0\right)}$ and ${b}_{g}^{\left(0\right)}$ were the learnable weight and bias vectors, respectively.

For the remaining layers, we used the same strategy as CAME, where we updated the embeddings of each node under the guidance of a heterogeneous graph and used a graph attention layer as the cell-type classifier. Finally, we used the cross-entropy loss with a label smoothing mechanism^50^ as the training loss and optimized by the Adam.^51^ In addition, we used the adjusted mutual information to select the best checkpoint, which was consistent with CAME. To evaluate the cell-type annotation performance, we adopted accuracy and macro-f1 as a metric. Each dataset pair underwent 20 distinct experiments using different seeds, and the average results from these experiments were used for comparison. Details of the cross-species datasets used in our study can be found in the Supplementary Methods.

#### GRN inference

This task involved the prediction of relationships among genes to gain insights into how genes work together to control cellular processes. We employed the DeepSEM framework^32^ as baseline in this task and then tested whether gene embeddings from the pre-defined models (GeneCompass, Geneformer, and scGPT) could benefit the output GRNs of DeepSEM. We applied each pre-trained model on Immune Human dataset^14^ to obtain gene embeddings. With similar tuning process above, we adjusted the learning rate and the number of training epochs in this task. We set the learning rate to 1e–4, the batch size to 64 and epochs to 40. We calculated the cosine similarity of gene embeddings as follows:

$$\cos \!{{\_}}{{\mbox{sim}}}=\frac{{x}_{i}\cdot {x}_{j}}{\left\Vert {x}_{i}\right\Vert \left\Vert {x}_{j}\right\Vert }$$

where ${x}_{i}$ and ${x}_{j}$ were gene-embedding vector and ||·|| denoted the Euclidean norm. We got gene–gene relationship based on cosine similarity by setting threshold to 0.4, which was optimized from 0 to 1. If the cosine similarity was above the threshold, there existed a relationship between the pair of genes. Then, we fed the log-transformed scRNA-seq expression data derived from the BEELINE framework^52^ after Z-normalization into DeepSEM. Moreover, we initialized MLPs by using the “kaiming_uniform”^4^ and initialized ${{\rm{W}}}$ by setting the matrix diagonal as zeros and the others following a Gaussian distribution ${{\rm{N}}}\left(\frac{1}{{{\rm{m}}}-1},{{{\rm{\varepsilon }}}}^{2}\right)$, in which m stands for a number of genes and ε denotes a small value to avoid being trapped in the local optimal. We applied ChIP-Seq data with variable TFs (Supplementary information, Table S5), generated from BEELINE framework^52^ as ground truth, and the values on the diagonal were fixed as zero in the entire training process to guarantee that W could obtain the regulatory network from learning procedure. Finally, we applied the gene relationships learned from the pre-defined models to update DeepSEM and then drew final GRNs. (Supplementary information, Fig. S6a).

#### Drug dose response prediction

This task referred to the relationship between drug dosage and the expression response of a gene. On the dataset provided by Srivatsan et al.,^53^ it could be described as ${{\rm{D}}}={\left\{{{{\rm{x}}}}_{{{\rm{i}}}},{{{\rm{d}}}}_{{{\rm{i}}}},{{{\rm{c}}}}_{{{\rm{i}}}}\right\}}_{{{\rm{i}}}=1}^{{{\rm{N}}}}$, where each ${{{\rm{x}}}}_{{{\rm{i}}}}\in {{{\rm{R}}}}^{{{\rm{G}}}}$ described the expression of G genes from cell i, if ${{{\rm{d}}}}_{{{\rm{id}}},{{\rm{i}}}}=0$, perturbation j was not applied to cell i. Unless stated otherwise, the sequel assumed the column vectors. Similarly, the vector of vectors ${{{\rm{c}}}}_{{{\rm{i}}}}=\left({{{\rm{c}}}}_{{{\rm{i}}}.1}\ldots ...{{{\rm{c}}}}_{{{\rm{i}}}.{{\rm{K}}}}\right)$ comprised additional discrete covariates such as cell types or species, where each covariate was itself a vector. Specifically, ${{{\rm{c}}}}_{{{\rm{i}}},{{\rm{j}}}}$ was a ${{{\rm{K}}}}_{{{\rm{j}}}}$-dimensional one-hot vector. We loaded the pre-trained CPA model and implemented CPA to predict the expression changes of specific gene (as an example, *MDM2*) in different drugs and dosage by following steps: (1) encoding the gene expression ${{{\rm{x}}}}_{{{\rm{i}}}}$ into an estimated basal state $\acute{{{\rm{z}}}}_{{{\rm{i}}}}^{{\mbox{basal}}}$ that did not contain any information about $\left({{{\rm{d}}}}_{{{\rm{i}}}},{{{\rm{c}}}}_{{{\rm{i}}}}\right)$, (2) combining $\acute{{{\rm{z}}}}_{{{\rm{i}}}}^{{\mbox{basal}}}$ with learnable embeddings about $\left({{{\rm{d}}}}_{{{\rm{i}}}},{{{\rm{c}}}}_{{{\rm{i}}}}\right)$, (3) employing gene embeddings generated by the pre-defined models (GeneCompass, Geneformer, and scGPT) to the Compositional Perturbation Autoencoder (CPA)^33^ model. (Supplementary information, Fig. S6b).

#### Gene expression profiling prediction

This task involved estimating the levels of gene expression based on various biological conditions. We employed the DeepCE^34^ model designed for phenotype-based compound screening to assess the impact of gene embeddings generated by the pre-defined models (GeneCompass, Geneformer, and scGPT) in gene expression profiles. The framework of this task consisted of three components: the first component included the feature transformation component (GCN), pre-trained network (human protein–protein interaction network, extracted from the STRING database^52^), and feed-forward neural network; the second component comprised an interaction network (multi-head attention); and the last component included a prediction network (two-layer feed-forward neural network with a rectified linear unit activation function).^34^ In the task, we set the learning rate to 2e–4, the batch size to 32 and epochs to 120. DeepCE captured features from the chemical compounds, L1000 genes,^54^ cells, dosage and then concatenated with gene embeddings collected from each pre-trained model in the first component. Moreover, high-level feature associations (features from L1000 genes and chemical compound) were generated in the second component. Furthermore, in the last component, all features learned from the previous components were concatenated as high-level features. Finally, we predicted the drug-induced changes in gene expression profiles (Supplementary information, Fig. S6c).

#### Gene dosage sensitivity predictions

Distinguishing between dosage-sensitive and dosage-insensitive transcription factors is critical for explaining CNVs in gene diagnosis. Traditional approaches used conservation and allele frequencies to predict dosage sensitivity. However, these characteristics did not vary with cell state and cannot capture specific tissues that would be influenced by dosage changes in the gene. Following the protocol of Geneformer,^15^ 10,000 random single-cell transcriptomes were used to fine-tune GeneCompass to distinguish between the dosage-sensitive and dosage-insensitive TFs.

#### In silico perturbation

GEARS learns gene embedding from a gene co-expression knowledge graph. This embedding was then combined with perturbation embedding from a GO-derived knowledge graph to predict post-perturbation expression. In our study, we replaced the GEARS gene embedding with GeneCompass gene embedding (Fig. 5a). We finetuned the model (epoch = 10) using the Norman^36^ dataset to predict gene expression on one- and two-gene perturbations. Since most genes do not exhibit significant variation between unperturbed and perturbed states, we utilized the MSE of the top 20 DEGs as the loss function for fine-tuning.

We evaluated the effect of GeneCompass on both one- and two-gene perturbations by holding out data of specific conditions during training. The mean expression changes of the experimental data were taken as the ground truth. We calculated the absolute difference between the predicted values and the ground-truth and selected top 20 genes with the most significant changes in the perturbed experiment to calculate the sum of the differences, which was defined as top 20 DEGs deviation as follows:

$${{\rm{Top}}}\, 20\, {{\rm{DEGs}}}^{\prime} \, {{\rm{deviation}}}={\sum }_{i=1}^{20}{|}{P}_{i}-{T}_{i}{|}$$

where ${P}_{i}$ was the predicted post-perturbation expression change for gene *i*, and ${T}_{i}$ was the ground truth of the mean expression change.

#### In silico quantitative perturbation

This task was designed to simulate cell reprogramming and differentiation. The perturbation state was characterized by cell and gene embeddings. The in silico quantitative knockout was performed by decreasing the target gene expression value in the single-cell transcriptome. The in silico overexpression was achieved by increasing the expression of the target gene to a specific level. Both in silico overexpression and knockout were performed as the preprocessing procedure before forwarding the single-cell transcriptome to GeneCompass. Notably, our method is capable of quantitative in silico overexpression or knockout by increasing or reducing gene expression to any value. Considering the batch effect of original and perturbed cells, a random cell sampling was employed to construct gene pairs for perturbation analysis. Especially for in silico knockout experiments, we only retained the original cells where the target genes were ranked in the top 50%, in order to guarantee the feasibility of in silico knockout.

After the manipulation of gene expression, the single-cell transcriptome was re-ranked based on the gene expression value. The post-perturbation cell embeddings were obtained from GeneCompass. The effect of in silico quantitative perturbation was measured by calculating the cosine similarity between the post-perturbation and ground-truth cell embeddings, which was defined as follows:

$${{\rm{sim}}}=\frac{1}{{{\rm{n}}}}{\sum }_{i=1}^{n}\left(\cos \!{\_} {{\rm{sim}}}\left({S}_{i}^{{\prime} },{T}_{i}\right)-\cos \_{{\rm{sim}}}\left({S}_{i},{T}_{i}\right)\right)$$

where ${S}_{i}$ and ${S}_{i}^{{\prime} }$ denoted the source and in silico perturbed cell embedding, and ${T}_{i}$ denoted the ground-truth cell embedding.

### Immunostaining of TF-overexpressing cells

Reprogramming of hESC and cultivation of gonadal progenitor-like cell HESCs were cultured on Matrigel-coated dishes in mTeSR plus (Stemcell Techonologies #100-0276). Y27623 was removed 24 h after passaging using ReLeSRTM (Stemcell Techonologies #100-0483), and the lentiviral infection was prepared after an additional 24 h. All overexpressed genes carrying *EF1α* promoter were constructed on lentiviral vectors purchased from VectorBuilder Inc. After overnight infection, mTeSR 1 fresh medium (Stemcell Techonologies #85850) was replaced daily. After 72 h, Minimum Essential Medium alpha (α-MEM, Invitrogen) containing 10% KSR (Gibco), 55 µM 2-mercaptoethanol (Gibco), and 100 U/mL penicillin/streptomycin (Gibco) was used for long-term cultivation. Other factors such as hSCF and GDNF should be added appropriately if necessary.

Cells stably overexpressing TFs were seeded on Matrigel-coated chamber slides (ThermoFisher Scientific #154534pk), and immunofluorescence was performed on day 3 after plating. The slides of cells with appropriate fusion degree were fixed with 4% PFA for 10 min at room temperature and washed with PBS three times for 5 min each time. Then TBST containing 10% of normal secondary antibody host serum was used to block unspecific antigens at room temperature for 1 h. According to the instructions, the primary antibody was diluted to an appropriate proportion with cell holding buffer (BioLegend #420201), and the cells were incubated at room temperature for 1 h. After PBS washing for three times with 5 min each, the secondary antibody was diluted to a concentration of 1:500 using cell holding buffer, and the cells were incubated at room temperature for 1 h. Then the cells were washed with PBS three times for five min each and sealed with an antifade mounting medium with DAPI (Beyotime #p0131). Leica Stellaris was used for shooting, and the saved LIF file was converted to TIF format for display by Leica application suite X.

To be noted, both homologous and non-homologous genes in the pretraining and full panel of gene-level tasks were encoded into the models for a fair comparison of GeneCompass with other models.

## Supplementary information

Supplementary information, Fig.S1

Supplementary information, Fig.S2

Supplementary information, Fig.S3

Supplementary information, Fig.S4

Supplementary information, Fig.S5

Supplementary information, Fig.S6

Supplementary information, Fig.S7

Supplementary information, Fig.S8

Supplementary information, Fig.S9

Supplementary information, Fig.S10

Supplementary information, Table S1

Supplementary information, Table S2

Supplementary information, Table S3

Supplementary information, Table S4

Supplementary information, Table S5

## Data Availability

The raw sequence data reported in this paper have been deposited in the Genome Sequence Archive in the National Genomics Data Center, China National Center for Bioinformation/Beijing Institute of Genomics, Chinese Academy of Sciences (GSA-Human: HRA008557) that are publicly accessible at https://ngdc.cncb.ac.cn/gsa-human. All codes including data preprocessing, model pre-training, multiple downstream tasks fine-tuning, and the corresponding examples were uploaded to GitHub repository: https://github.com/xCompass-AI/GeneCompass.
