# Supplementary material for: GeneCompass: deciphering universal gene regulatory mechanisms with a knowledge-informed cross-species foundation model
Source: Cell Res. 2024 Oct 8;34(12):830–45. doi: 10.1038/s41422-024-01034-y (PMC11615217; doi:10.1038/s41422-024-01034-y)
Supplement: Supplementary file 1 — Supplementary information, Fig.S1 [file 41422_2024_1034_MOESM1_ESM.pdf]

a

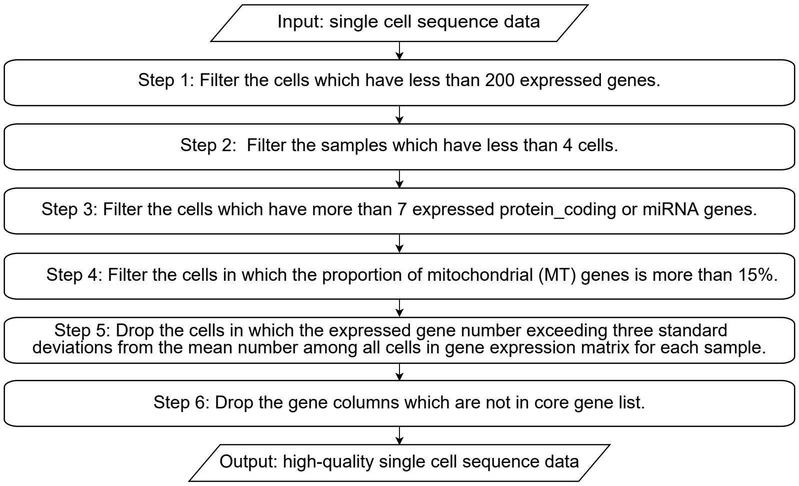

b

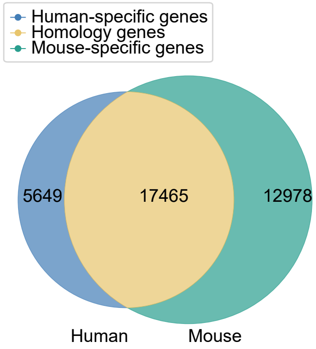

c

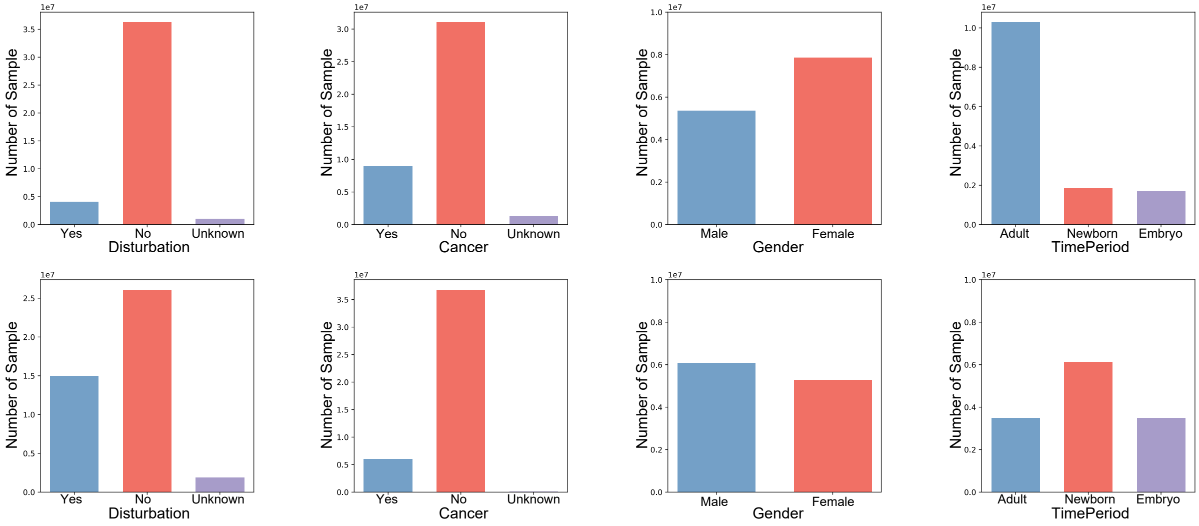

d

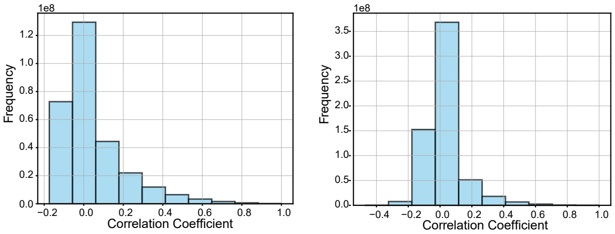

e

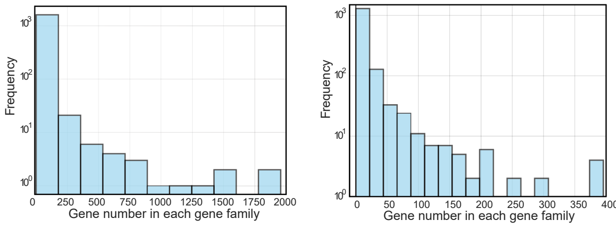

f

	E11.5	E12.5	E13.5	E14.5	E15.5	E16.5	P0	Adult8wks
Bcell(CD19+)								275966
Bcell(CD43-)								280869
Cerebellum								289862
Cerebrum								297853
FatPad								241948
GenitalFatPad								258323
Heart	240011	242688	236483	229637	237521	240511	241983	271809
Kidney				226915	230444	229026	243531	285957
Liver	292502	307310	300688	292122	309640	312281	308426	291265
Lung				222078	211094	233942	257318	269387
Mel								293505
SkMuscle								224565
Spleen								284852
Thymus								250141
Tnaive								262856
Face	203800	208538	206551	221499	219099			
Forebrain	218477	232475	244758	239726	239221	243938	261127	
Hindbrain	231394	242893	268910	252662	269436	287536	279773	
Intestine				222825	266380	274507	272316	
Limb	199584	196687	212783	215882	211027			
Midbrain	227072	226485	253758	239601	264915	257886	256837	
NeuralTube	230877	248336	280886	275683	281323			
Stomach				213747	228327	230716	263349	
416b								263107
A20B								264741
Lgint								257749

Fig. S1| Preprocessing of multi-species training data and statistics of prior knowledge. a, Preprocessing flowchart for scCompass-126M. **b,** Illustration of diversity from single-cell perturbations, cancer cell identification, gender distribution, and cell differentiation time for humans (first row) and mice (second row). **c,** Number of homologous, human-specific, and mouse-specific genes in the token dictionary. **d,** Histogram of the Pearson correlation coefficient for all gene pairs in humans (left) and mice (right). **e,** Histogram of gene numbers in each gene family for humans (left) and mice (right). **f,** Multi-tissue developmental stage samples in mouse ENCODE and the number of PECA2 TF-TG edges in each mouse GRN.
