# Supplementary material for: GeneCompass: deciphering universal gene regulatory mechanisms with a knowledge-informed cross-species foundation model
Source: Cell Res. 2024 Oct 8;34(12):830–45. doi: 10.1038/s41422-024-01034-y (PMC11615217; doi:10.1038/s41422-024-01034-y)
Supplement: Supplementary file 2 — Supplementary information, Fig.S2 [file 41422_2024_1034_MOESM2_ESM.pdf]

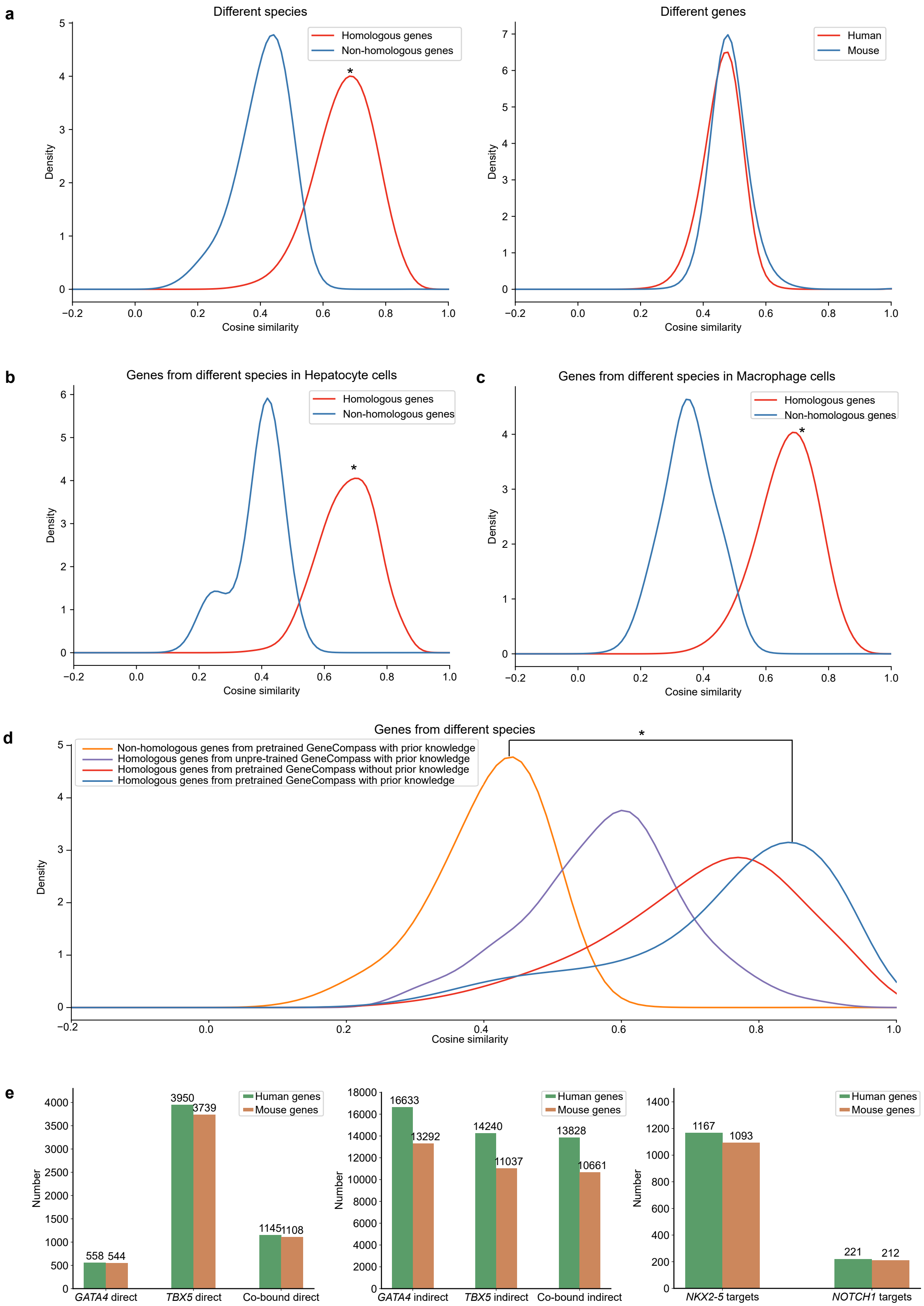
a
Different species
Homologous genes
Non-homologous genes
Density
Cosine similarity
Different genes
Human
Mouse
Density
Cosine similarity
b
Genes from different species in Hepatocyte cells
Homologous genes
Non-homologous genes
Density
Cosine similarity
c
Genes from different species in Macrophage cells
Homologous genes
Non-homologous genes
Density
Cosine similarity
d
Genes from different species
Non-homologous genes from pretrained GeneCompass with prior knowledge
Homologous genes from unpre-trained GeneCompass with prior knowledge
Homologous genes from pretrained GeneCompass without prior knowledge
Homologous genes from pretrained GeneCompass with prior knowledge
Density
Cosine similarity
e
Human genes
Mouse genes
Number
558
544
3950
3739
1145
1108
GATA4 direct
TBX5 direct
Co-bound direct
16633
13292
14240
11037
13828
10661
GATA4 indirect
TBX5 indirect
Co-bound indirect
1167
1093
221
212
NKX2-5 targets
NOTCH1 targets

Fig. S2| Analysis of gene embedding and statistics of corresponding genes. a, Cosine similarity among different genes in the same species (left). Cosine similarity between homologous and non-homologous genes of different species (right). A more generalizable experiment were performed without restricting to specific cell types, including GABAergic neuron, Cardiomyocyte, Fibroblast, Erythroblast, and etc. **b**, Cosine similarity between homologous and non-homologous genes in Hepatocyte cells of different species. **c**, Cosine similarity between homologous and non-homologous genes in Macrophage cells of different species. **d**, Comparison among cosine similarity of non-homologous gene embeddings from the pretrained GeneCompass with prior knowledge and homologous gene embeddings which came from pretrained GeneCompass with prior knowledge, pretrained GeneCompass without prior knowledge, and un-pretrained GeneCompass with prior knowledge. **e**, Statistics of genes in the deletion of *GATA4* and *TBX5*. Mouse genes are obtained by homologous mapping. (*$P < 0.05$ wilcoxon-test, NS no significance)
