# Supplementary material for: GeneCompass: deciphering universal gene regulatory mechanisms with a knowledge-informed cross-species foundation model
Source: Cell Res. 2024 Oct 8;34(12):830–45. doi: 10.1038/s41422-024-01034-y (PMC11615217; doi:10.1038/s41422-024-01034-y)
Supplement: Supplementary file 3 — Supplementary information, Fig.S3 [file 41422_2024_1034_MOESM3_ESM.pdf]

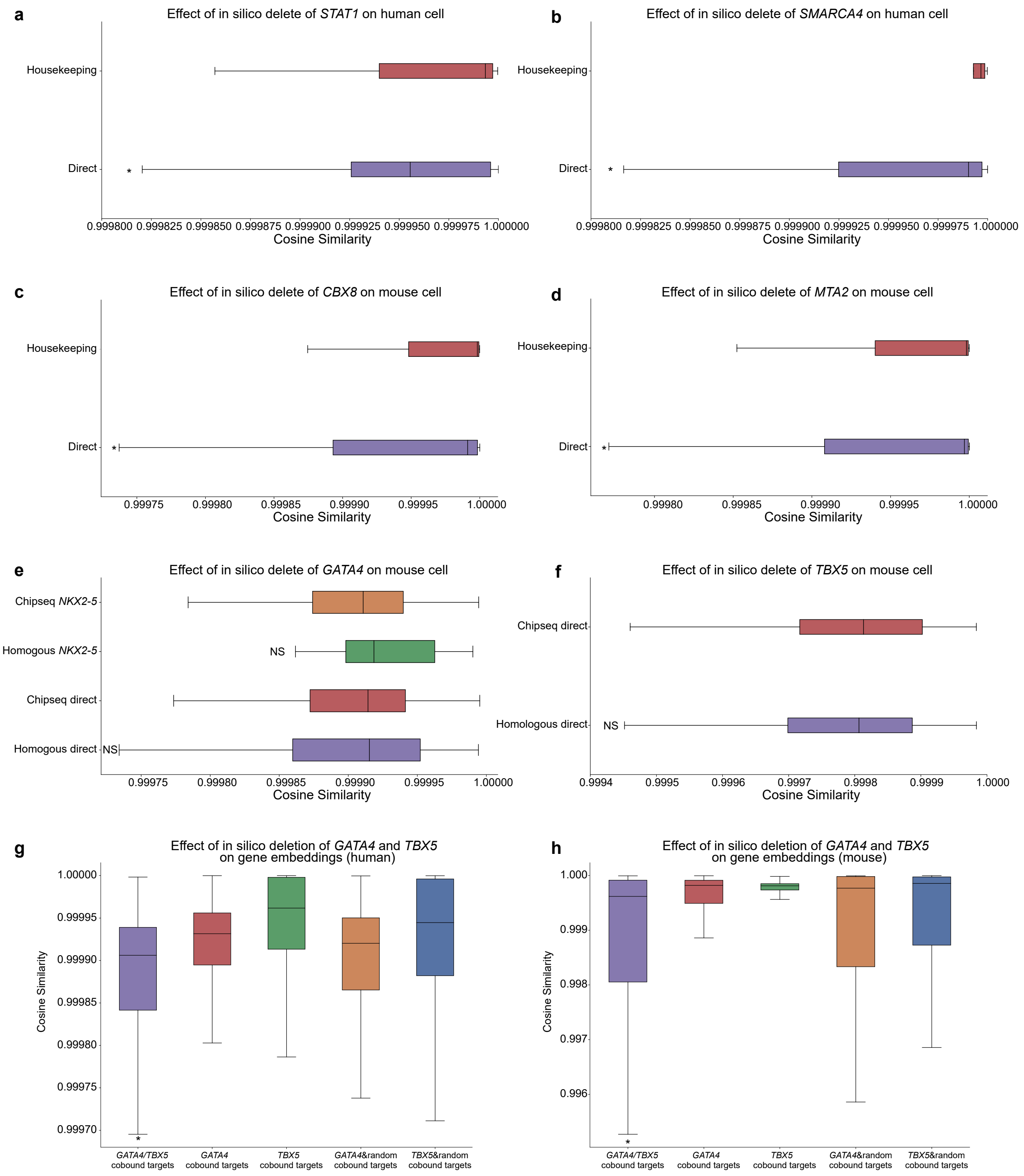

Fig. S3| Analysis of gene embedding. a, Comparison of the effects of *in silico* deletion of *STAT1* on housekeeping genes and direct target genes in human PBMC cells. **b,** Comparison of the effects of *in silico* deletion of *SMARCA4* on housekeeping genes and direct target genes in human acute myeloid leukemia cells. **c,** Comparison of the effects of *in silico* deletion of *CBX5* on housekeeping genes and direct target genes in mouse embryonic stem cells. **d,** Comparison of the effects of *in*

silico deletion of *MTA2* on mouse housekeeping genes and direct target genes in colonic epithelium cells. **e**, Effects of in silico deletion of *GATA4* cells on different sources of direct targets and *NKX2-5* targets, including ChIP-Seq ground-truth and homologous mapping from humans. **f,** Effects of in silico deletion of *TBX5* cells on different sources of direct targets, including ChIP-Seq ground-truth and homologous mapping from humans. **g-h,** Effects of the individual and combined deletion of *GATA4* and *TBX5* as well as their combinatorial deletion with other genes that are not known to co-bind with the co-bound target genes in humans and mice. (*$P < 0.05$ wilcoxon-test, NS no significance)
