# Supplementary material for: GeneCompass: deciphering universal gene regulatory mechanisms with a knowledge-informed cross-species foundation model
Source: Cell Res. 2024 Oct 8;34(12):830–45. doi: 10.1038/s41422-024-01034-y (PMC11615217; doi:10.1038/s41422-024-01034-y)
Supplement: Supplementary file 4 — Supplementary information, Fig.S4 [file 41422_2024_1034_MOESM4_ESM.pdf]

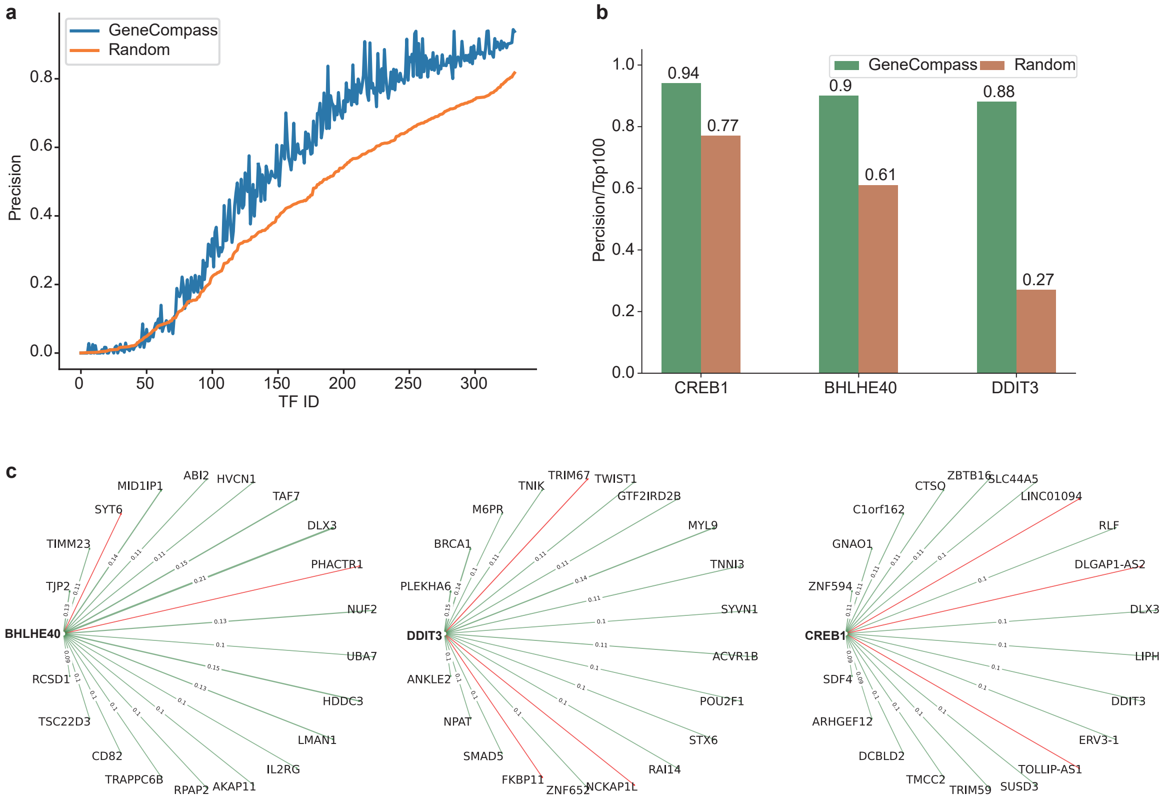

a
GeneCompass
Random
Precision
0.0
0.2
0.4
0.6
0.8
0
50
100
150
200
250
300
TF ID
b
GeneCompass
Random
Percision/Top100
0.0
0.2
0.4
0.6
0.8
1.0
0.94
0.77
0.9
0.61
0.88
0.27
CREB1
BHLHE40
DDIT3
c
BHLHE40
MID1IP1
ABI2
HVCN1
TAF7
SYT6
DLX3
TIMM23
PHACTR1
TJP2
NUF2
UBA7
RCSD1
HDDC3
TSC22D3
LMAN1
CD82
IL2RG
TRAPPC6B
RPAP2
AKAP11
DDIT3
TRIM67
TWIST1
TNIK
GTF2IRD2B
M6PR
MYL9
BRCA1
TNNI3
PLEKHA6
SYVN1
ACVR1B
ANKLE2
POU2F1
NPAT
STX6
SMAD5
RAI14
FKBP11
ZNF652
NCKAP1L
CREB1
ZBTB16
SLC44A5
CTSO
LINC01094
C1orf162
RLF
GNAO1
DLGAP1-AS2
ZNF594
DLX3
LIPH
SDF4
DDIT3
ARHGEF12
ERV3-1
DCBLD2
TOLLIP-AS1
TMCC2
SUSD3
TRIM59

Fig. S4| Evaluation on GeneCompass's ability in GRN recognition. a, Comparison of GRN inference accuracy between GeneCompass (*in silico* deletion) and random sampling. **b**, Comparison of precision for the top 100 most likely predicted target genes (TGs) between GeneCompass and random selection after perturbing CREB1, BLHE40, and DDIT3, which intersected with the ChIP-Atlas ground-truth data. **c**, Illustration of the top 20 most likely TGs predicted by GeneCompass after perturbing CREB1, BLHE40, and DDIT3, with 17, 18, and 17 genes overlapping with TGs identified for each TF in ChIP-Atlas. Green lines indicate correct predictions, while red lines indicate incorrect predictions.
