# Supplementary material for: GeneCompass: deciphering universal gene regulatory mechanisms with a knowledge-informed cross-species foundation model
Source: Cell Res. 2024 Oct 8;34(12):830–45. doi: 10.1038/s41422-024-01034-y (PMC11615217; doi:10.1038/s41422-024-01034-y)
Supplement: Supplementary file 5 — Supplementary information, Fig.S5 [file 41422_2024_1034_MOESM5_ESM.pdf]

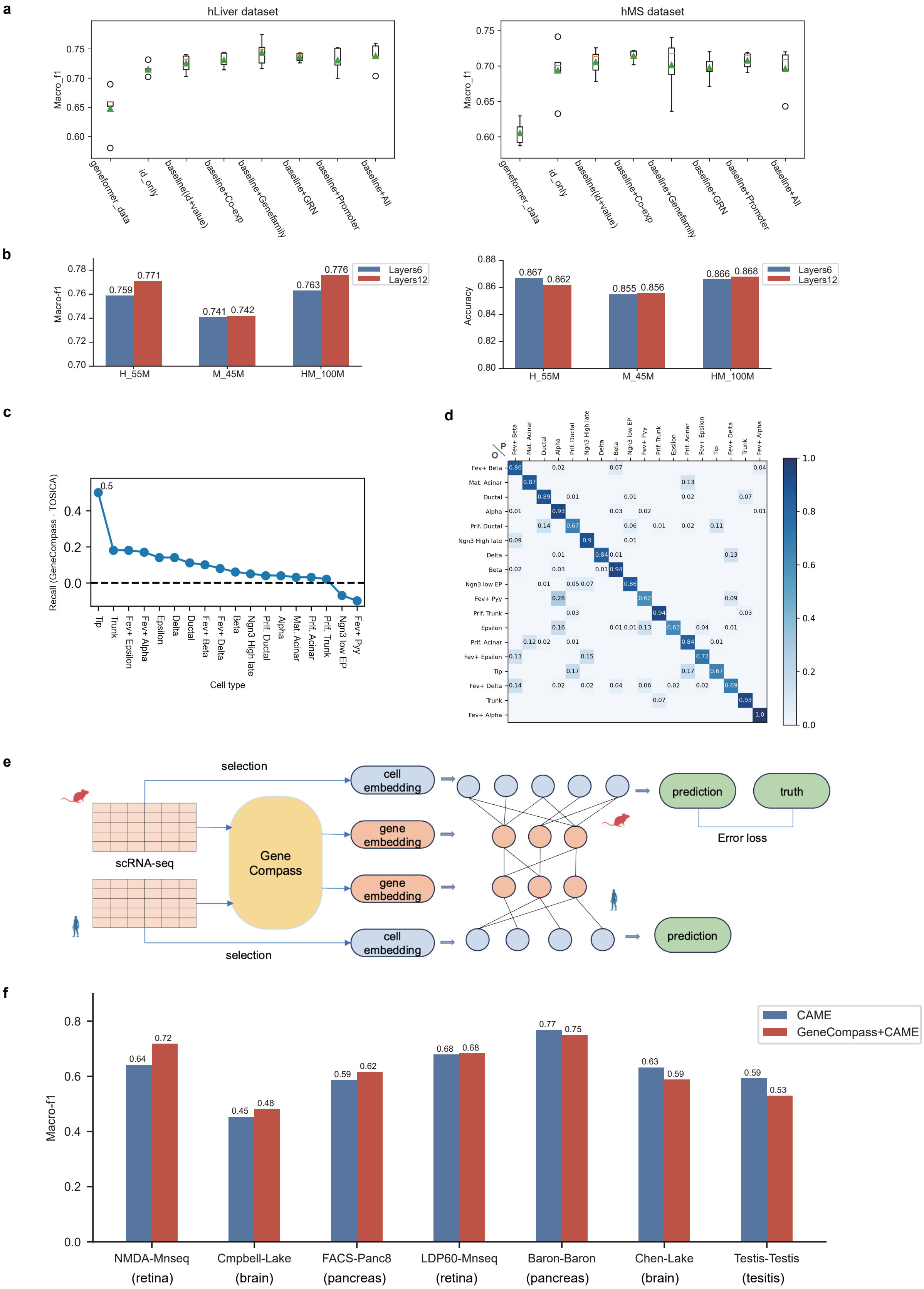
a
hLiver dataset
hMS dataset
Macro_f1
geneformer_data
id_only
baseline(id+value)
baseline+Co-exp
baseline+Genefamily
baseline+GRN
baseline+Promoter
baseline+All
b
Macro-f1
Accuracy
Layers6
Layers12
H_55M
M_45M
HM_100M
0.759
0.771
0.741
0.742
0.763
0.776
0.867
0.862
0.855
0.856
0.866
0.868
c
Recall (GeneCompass - TOSICA)
0.5
Cell type
Tip
Trunk
Fev+ Epsilon
Fev+ Alpha
Epsilon
Delta
Ductal
Fev+ Beta
Fev+ Delta
Beta
Ngn3 High late
Prlf. Ductal
Alpha
Mat. Acinar
Prlf. Acinar
Prlf. Trunk
Ngn3 low EP
Fev+ Pyy
d
O
P
Fev+ Beta
Mat. Acinar
Ductal
Alpha
Prlf. Ductal
Ngn3 High late
Delta
Beta
Ngn3 low EP
Fev+ Pyy
Prlf. Trunk
Epsilon
Prlf. Acinar
Fev+ Epsilon
Tip
Fev+ Delta
Trunk
Fev+ Alpha
e
selection
scRNA-seq
Gene Compass
cell embedding
gene embedding
prediction
truth
Error loss
f
Macro-f1
CAME
GeneCompass+CAME
NMDA-Mnseq (retina)
Cmpbell-Lake (brain)
FACS-Panc8 (pancreas)
LDP60-Mnseq (retina)
Baron-Baron (pancreas)
Chen-Lake (brain)
Testis-Testis (tesitis)
0.64
0.72
0.45
0.48
0.59
0.62
0.68
0.68
0.77
0.75
0.63
0.59
0.59
0.53

**Fig. S5| Prior knowledge ablation and results of cell type annotation. a,** The ablation study of four kinds of prior knowledges used for GeneCompass in the hMS dataset, where “+All” implies that all prior knowledges are concatenated. **b**, Ablation study of GeneCompass transformer layers in the hMS dataset. “layers6” and “layers12” denote self-attention transformer with 6 layers and 12 layers, respectively. **c**, Differences in recall between GeneCompass and TOSICA for different cell types in the mPancreas dataset. **d,** GeneCompass confusion matrix on the mPancreas dataset. **e,** Gene embedding of GeneCompass is integrated into CAME for cross-species cell-type annotation. **f**, The macro-f1 of cross-species cell-type annotation from mouse to human by using GeneCompass+CAME and CAME.
