# Supplementary material for: GeneCompass: deciphering universal gene regulatory mechanisms with a knowledge-informed cross-species foundation model
Source: Cell Res. 2024 Oct 8;34(12):830–45. doi: 10.1038/s41422-024-01034-y (PMC11615217; doi:10.1038/s41422-024-01034-y)
Supplement: Supplementary file 6 — Supplementary information, Fig.S6 [file 41422_2024_1034_MOESM6_ESM.pdf]

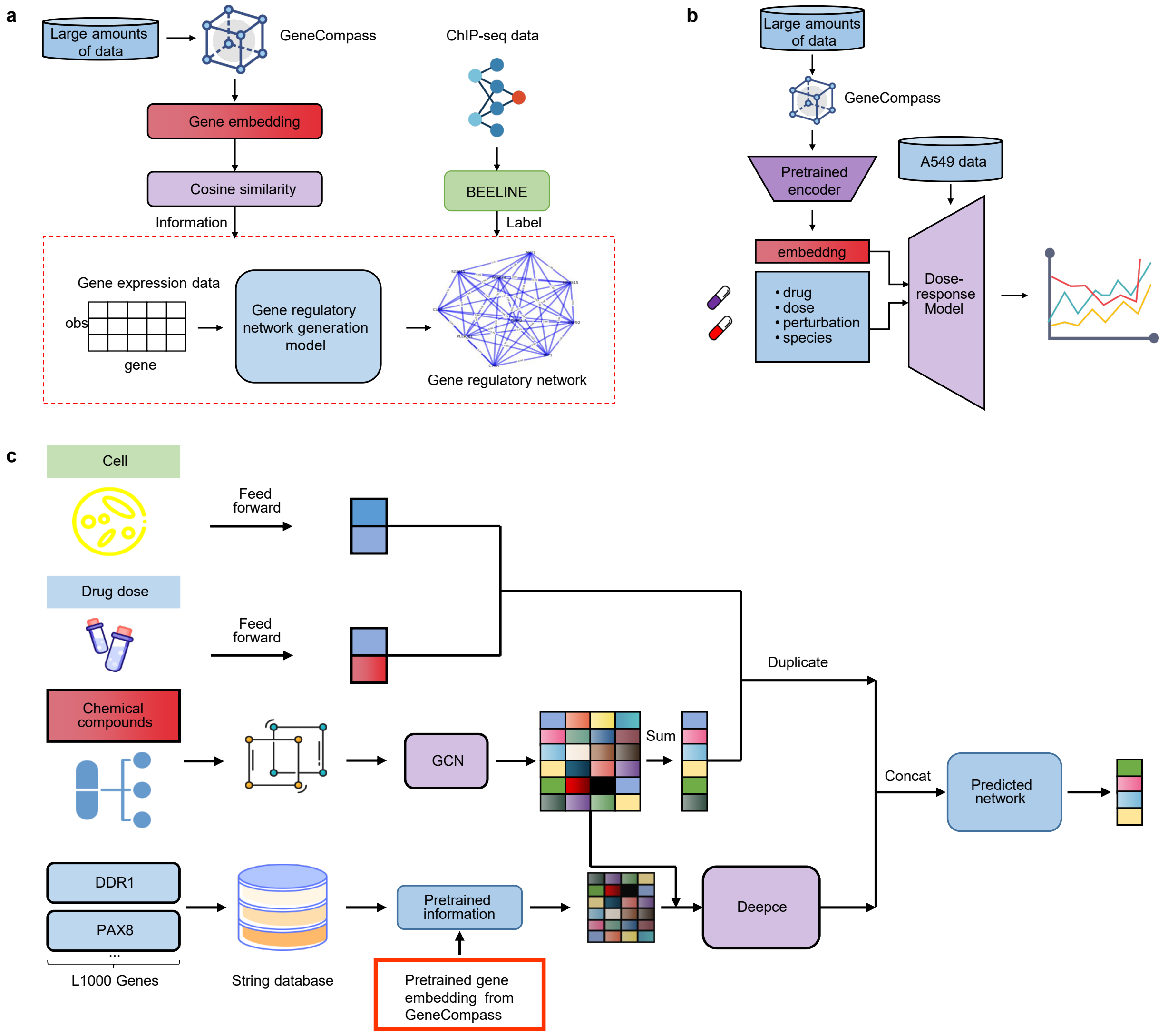

Fig. S6| Workflows of three downstream tasks. a, GRN inference, gene-gene relationship information captured by GeneCompass is used, and the ground truth comes from ChIP-Seq data. **b,** The detailed process of durg dose response prediction task. **c,** Gene expression profile prediction. Combined features of cell, drug, chemical compounds and genes are used to predict gene expression.
