# Supplementary material for: GeneCompass: deciphering universal gene regulatory mechanisms with a knowledge-informed cross-species foundation model
Source: Cell Res. 2024 Oct 8;34(12):830–45. doi: 10.1038/s41422-024-01034-y (PMC11615217; doi:10.1038/s41422-024-01034-y)
Supplement: Supplementary file 7 — Supplementary information, Fig.S7 [file 41422_2024_1034_MOESM7_ESM.pdf]

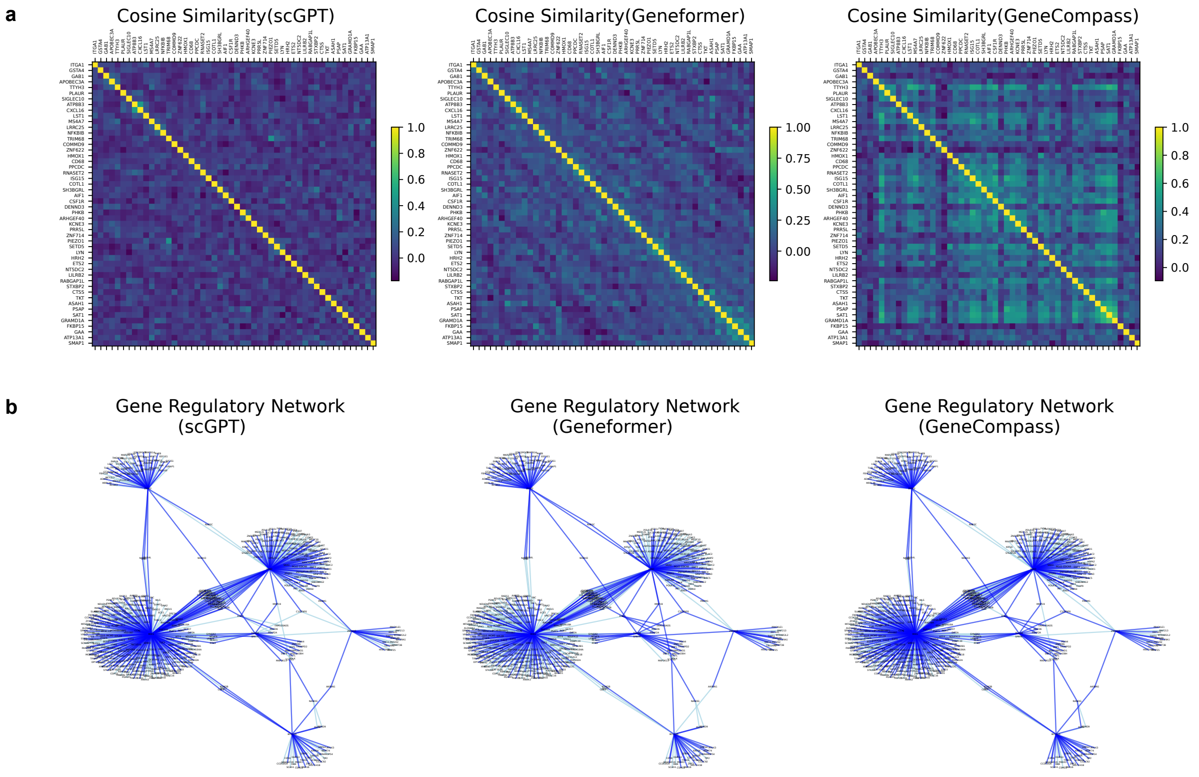

c

Model	DeepSEM	scGPT	Genformer	GeneCompass -0.05M	GeneCompass- 0.5M	GeneCompass- 5M	GeneCompass- 55M
AUPRC	0.113	0.112	0.109	0.116	0.117	0.117	0.121

d

Cell	Condition	scGPT	Genformer	GeneCompass- 0.05M	GeneCompass- 0.5M	GeneCompass -5M	GeneCompass- 55M
A549	SAHA	0.93	0.89	0.88	0.94	0.95	0.94
A549	Nutlin	0.85	0.89	0.63	0.65	0.70	0.88
A549	Dex	0.80	0.94	0.88	0.84	0.84	0.90
A549	Average	0.86	0.907	0.797	0.81	0.83	0.907

Fig. S7| GRN inference and drug dose response prediction results obtained using different models. a, From left to right, the graphs display the cosine similarity of gene embeddings computed by scGPT, Geneformer, and GeneCompass, respectively. Within each graph, the horizontal and vertical axes denote the names of genes. **b,** From left to right, the figures illustrate the GRN inference results generated by scGPT, Geneformer, and GeneCompass. Within these illustrations, all lines depict the model's predictions, while the dark blue lines correspond to the true positive results. **c,** The performance of each model in the GRN inference task. **d,** Performance

of each model in the drug dose–response prediction task. Specifically, GeneCompass-0.05M, GeneCompass-0.5M, GeneCompass-5M, and GeneCompass-55M correspond to the GeneCompass models trained by 0.05 million, 0.5 million, 5 million, and 55 million human data, respectively.
