# Supplementary material for: GeneCompass: deciphering universal gene regulatory mechanisms with a knowledge-informed cross-species foundation model
Source: Cell Res. 2024 Oct 8;34(12):830–45. doi: 10.1038/s41422-024-01034-y (PMC11615217; doi:10.1038/s41422-024-01034-y)
Supplement: Supplementary file 8 — Supplementary information, Fig.S8 [file 41422_2024_1034_MOESM8_ESM.pdf]

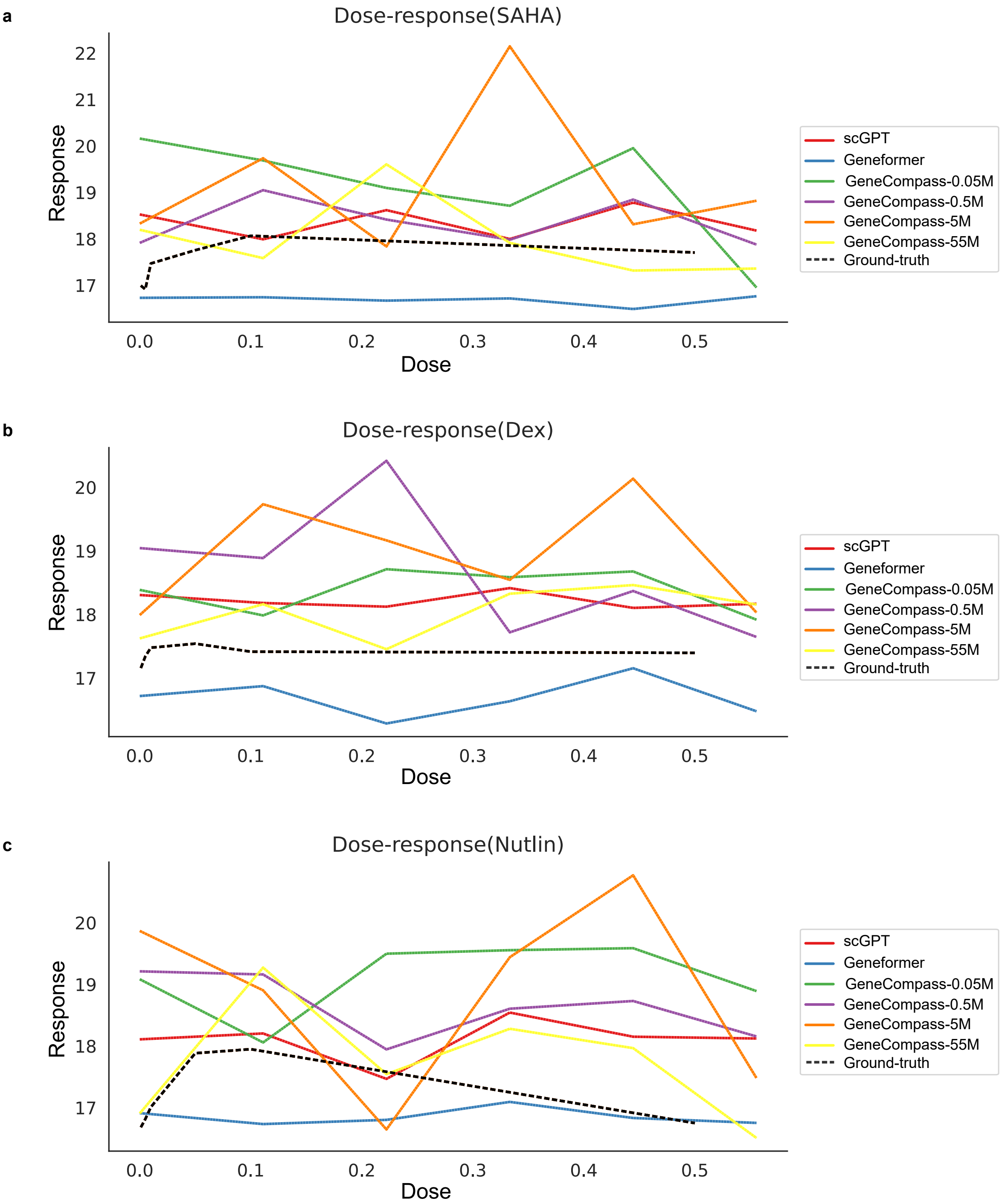

Fig. S8| Results of drug dose-response prediction task. All solid lines in the figure represent the predictive responses of a model when exposed to drugs: **a,** SAHA; **b,** Dex; **c,** Nutlin. Specifically, GeneCompass-0.05M, GeneCompass-0.5M, GeneCompass-5M, and GeneCompass-55M correspond to the GeneCompass models trained by 0.05 million, 0.5 million, 5 million, and 55 million human data, respectively. The dashed lines "--" denote the ground-truth results.
