# Supplementary material for: GeneCompass: deciphering universal gene regulatory mechanisms with a knowledge-informed cross-species foundation model
Source: Cell Res. 2024 Oct 8;34(12):830–45. doi: 10.1038/s41422-024-01034-y (PMC11615217; doi:10.1038/s41422-024-01034-y)
Supplement: Supplementary file 9 — Supplementary information, Fig.S9 [file 41422_2024_1034_MOESM9_ESM.pdf]

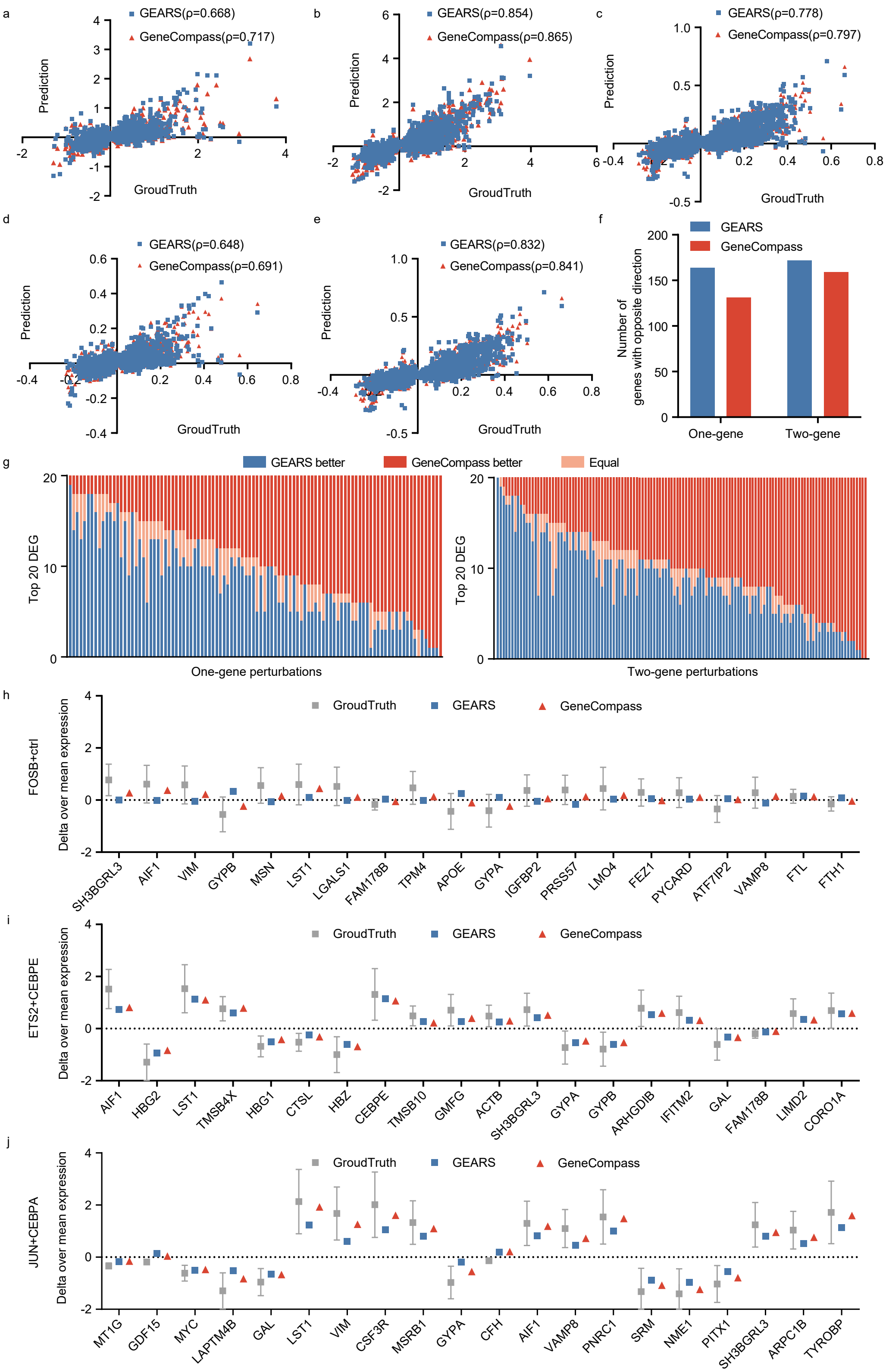

a
GEARS(ρ=0.668)
GeneCompass(ρ=0.717)
Prediction
GroudTruth
b
GEARS(ρ=0.854)
GeneCompass(ρ=0.865)
Prediction
GroudTruth
c
GEARS(ρ=0.778)
GeneCompass(ρ=0.797)
Prediction
GroudTruth
d
GEARS(ρ=0.648)
GeneCompass(ρ=0.691)
Prediction
GroudTruth
e
GEARS(ρ=0.832)
GeneCompass(ρ=0.841)
Prediction
GroudTruth
f
GEARS
GeneCompass
Number of genes with opposite direction
One-gene
Two-gene
g
GEARS better
GeneCompass better
Equal
Top 20 DEG
One-gene perturbations
Two-gene perturbations
h
GroudTruth
GEARS
GeneCompass
FOSB+ctrl
Delta over mean expression
SH3BGRL3
AIF1
VIM
GYPB
MSN
LST1
LGALS1
FAM178B
TPM4
APOE
GYPA
IGFBP2
PRSS57
LMO4
FEZ1
PYCARD
ATF7IP2
VAMP8
FTL
FTH1
i
GroudTruth
GEARS
GeneCompass
ETS2+CEBPE
Delta over mean expression
AIF1
HBG2
LST1
TMSB4X
HBG1
CTSL
HBZ
CEBPE
TMSB10
GMFG
ACTB
SH3BGRL3
GYPA
GYPB
ARHGDIB
IFITM2
GAL
FAM178B
LIMD2
CORO1A
j
GroudTruth
GEARS
GeneCompass
JUN+CEBPA
Delta over mean expression
MT1G
GDF15
MYC
LAPTM4B
GAL
LST1
VIM
CSF3R
MSRB1
GYPA
CFH
AIF1
VAMP8
PNRC1
SRM
NME1
PITX1
SH3BGRL3
ARPC1B
TYROBP

Fig. S9| *In silico* gene perturbation prediction. Scatter plots of the predicted and true changes in gene expression for **a,** one-gene perturbations and **b,** two-gene perturbations. Scatter plots of the predicted and true log fold changes in gene expression for **c,** all perturbations**, d,** one-gene perturbations and **e,** two-gene perturbations. Each dot represents a specific gene, and Spearman's correlation coefficient is marked as "ρ".**f,** Total number of the top 20 DEGs where the predicted post perturbation differential expression is in the incorrect direction of the ground truth for one-gene and two-gene perturbations. **g,** Comparison between GeneCompass and GEARS for the prediction of the top 20 genes in one-gene perturbations and two-gene perturbations. "GeneCompass better" is defined as GeneCompass having a smaller deviation than GEARS for a specific gene. Expression changes predicted by GeneCompass and GEARS for perturbations of **h,** FOSB+ control, **i,** EST2+CEBPE, **j,** JUN+CEBPA.
