# Supplementary material for: GeneCompass: deciphering universal gene regulatory mechanisms with a knowledge-informed cross-species foundation model
Source: Cell Res. 2024 Oct 8;34(12):830–45. doi: 10.1038/s41422-024-01034-y (PMC11615217; doi:10.1038/s41422-024-01034-y)
Supplement: Supplementary file 10 — Supplementary information, Fig.S10 [file 41422_2024_1034_MOESM10_ESM.pdf]

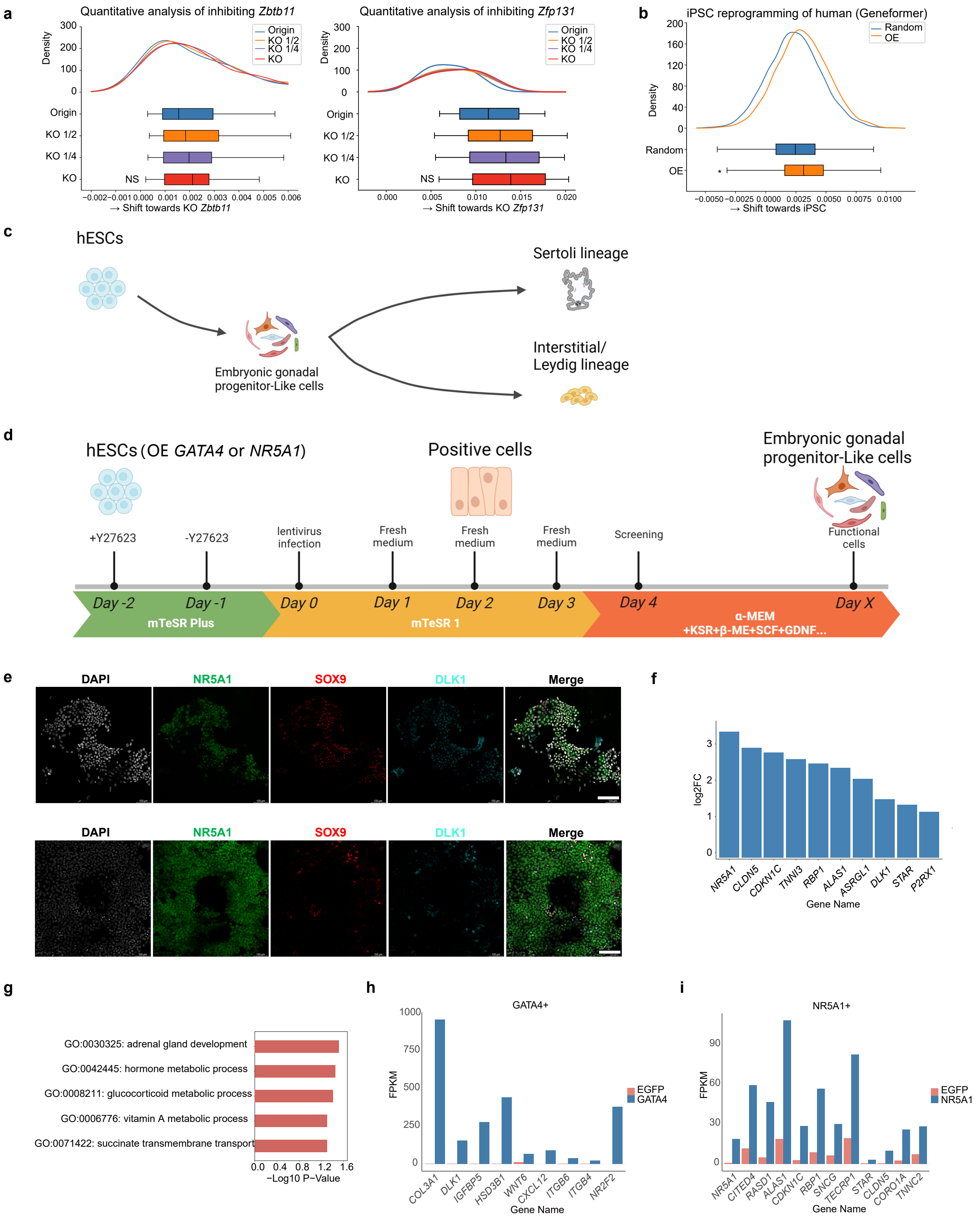

Fig. S10| GeneCompass predicts core factors for ESC differentiation into gonadal lineage and validation. a, Perturbation of ESCs by *in silico* knocking out *Zbtb11* or *Zfp131* to reach the

Endoderm state in mouse cells. “KO 1/2” denotes reducing the target gene to the half, “KO 1/4” denotes reducing *Zbtb11* or *Zfp131* gene expression to its quarter; and “KO” reducing fully knocking out *Zbtb11* or *Zfp131* gene in the cell. **b**, *In silico* overexpression of OSKM in human fibroblasts using Geneformer. *In silico* overexpression of four other random genes is used as control. In each simulation group, all embedding pairs between perturbed fibroblast cells and iPSCs are used to calculate the cosine similarity. The cosine similarity of all pairs in each group is simultaneously presented using probability density and box plots. **c,** Diagram of inducing differentiation of ESCs into gonadal cells. **d,** Diagram illustrating the protocol for differentiation of ESCs into gonadal progenitor-like cells *in vitro*. **e,** Protein co-immunofluorescence staining for markers of Sertoli (SOX9, red), interstitial/Leydig lineage (DLK1, cyan), and NR5A1^{+} cells (NR5A1: EGFP reporter). Scale bar: 100 μm. **f,** The identification of upregulated gonadal lineage-related marker genes in the *NR5A1* overexpression group compared to cells derived from wild type ESCs, with fold changes exceeding 2-fold. **g,** Gene ontology enrichment analysis was performed using DAVID for the total up-regulated genes with a two-fold change in the *NR5A1* overexpression group compared to cells derived from wild-type ESCs. **h-i** *FPKM* values of gonadal development related genes after overexpression of GATA4 (g) and *NR5A1* (h) in hESC alone. As shown in the figure, compared with the EGFP control group, the single overexpression of *GATA4* and *NR5A1* significantly increased a series of gonadal development related genes respectively. For example, *DLK1*, *WNT6*, and *NR2F2*, which play an important role in gonadal development, are significantly up-regulated in GATA4+ cells. However, NR5A1^{+} cells significantly up-regulated the representative genes directly involved in gonadal development, such as *STAR*, *ALAS1*, *RBP1*, etc.
