# Supplementary material for: GeneCompass: deciphering universal gene regulatory mechanisms with a knowledge-informed cross-species foundation model
Source: Cell Res. 2024 Oct 8;34(12):830–45. doi: 10.1038/s41422-024-01034-y (PMC11615217; doi:10.1038/s41422-024-01034-y)
Supplement: Supplementary file 11 — Supplementary information, Table S1 [file 41422_2024_1034_MOESM11_ESM.pdf]

Table S1. The cell number of each resource. The dataset contains 101,768,420 single cells, consisting of 53,568,337 human cells and 48,200,083 mouse cells.

Species	Source				Summation
	NCBI	EBI	CNCB	CELLxGENE	
Human	38,052,444	9,828,770	0	5,687,123	53,568,337
Mouse	41,975,962	4,083,401	2,140,720	0	48,200,083
