# Supplementary material for: GeneCompass: deciphering universal gene regulatory mechanisms with a knowledge-informed cross-species foundation model
Source: Cell Res. 2024 Oct 8;34(12):830–45. doi: 10.1038/s41422-024-01034-y (PMC11615217; doi:10.1038/s41422-024-01034-y)
Supplement: Supplementary file 12 — Supplementary information, Table S2 [file 41422_2024_1034_MOESM12_ESM.pdf]

Table S2. The impact of prior knowledge on the performance of multiple downstream tasks. GeneCompass were pre-trained using the full human dataset (~55 millions) and further finetuned on multiple downstream tasks, including cell type annotation, dosage sensitive TF classification, GRN inference, drug dose response prediction, and gene expression profiling. In the fine-tuning stage, 5-fold cross validation was performed. The best results for each task are marked in red.

GeneCompass		Cell type annotation hMS		Cell type annotation hLiver		Dosage sensitive TF classification	GRN inference	Drug dose response	Gene expression profiling
		Macro-f1	Accuracy	Macro-f1	Accuracy	AUC	AUPRC	R2	RMSE
w/o prior	baseline (id+value)	0.726±0.016	0.835±0.011	0.725±0.007	0.810±0.004	0.916±0.029	0.114±0.009	0.797±0.004	2.00955
w/ prior	baseline + Co-exp	0.743±0.030	0.848±0.009	0.73±0.013	0.807±0.008	0.894±0.018	0.117±0.012	0.800±0.003	2.00953
	baseline + Genefamily	0.734±0.019	0.856±0.007	0.734±0.028	0.810±0.018	0.907±0.048	0.116±0.011	0.763±0.010	2.00953
	baseline + GRN	0.728±0.015	0.836±0.011	0.737±0.012	0.811±0.011	0.944±0.027	0.117±0.008	0.853±0.006	2.00949
	baseline + Promoter	0.737±0.044	0.849±0.013	0.735±0.027	0.818±0.02	0.910±0.014	0.116±0.010	0.847±0.003	2.00947
	baseline + All	0.748±0.034	0.856±0.003	0.746±0.008	0.817±0.008	0.950±0.023	0.120±0.005	0.877±0.002	2.00944
