# Supplementary material for: GeneCompass: deciphering universal gene regulatory mechanisms with a knowledge-informed cross-species foundation model
Source: Cell Res. 2024 Oct 8;34(12):830–45. doi: 10.1038/s41422-024-01034-y (PMC11615217; doi:10.1038/s41422-024-01034-y)
Supplement: Supplementary file 13 — Supplementary information, Table S3 [file 41422_2024_1034_MOESM13_ESM.pdf]

Table S3. The impact of input data and absolute gene expression values on the performance of multiple downstream tasks. Considering that pre-training on the whole datasets would cost much time, we built two small datasets of 5 million single cells by randomly selecting from Geneformer and GeneCompass corpus. Then, Geneformer and GeneCompass were pre-trained based on the corresponding dataset and further finetuned on multiple downstream tasks, including cell type annotation, dosage sensitive TF classification, GRN inference, drug dose response prediction, and gene expression profiling. In the fine-tuning stage, 5-fold cross validation was performed. The best results for each task are marked in red.

Method		Cell type annotation (hMS)		Cell type annotation (hLiver)		Dosage sensitive TF classification	GRN inference	Drug dose response	Gene expression profiling
		Macro-f1	Accuracy	Macro-f1	Accuracy	AUC	AUPRC ratio	R2	RMSE
Geneformer		0.605±0.017	0.77±0.031	0.648±0.041	0.749±0.025	0.88±0.025	0.97±0.029	0.83±0.000	2.02
GeneCompass	Id only	0.694±0.039	0.838±0.014	0.715±0.011	0.797±0.017	0.93±0.034	0.96±0.021	0.73±0.001	2.02
	baseline (id+value)	0.706±0.019	0.829±0.017	0.726±0.016	0.807±0.013	0.88±0.032	1.10±0.005	0.78±0.002	2.02
	baseline + all prior	0.696±0.031	0.826±0.01	0.738±0.022	0.802±0.006	0.94±0.031	1.10±0.038	0.75±0.004	2.02
