# Supplementary material for: GeneCompass: deciphering universal gene regulatory mechanisms with a knowledge-informed cross-species foundation model
Source: Cell Res. 2024 Oct 8;34(12):830–45. doi: 10.1038/s41422-024-01034-y (PMC11615217; doi:10.1038/s41422-024-01034-y)
Supplement: Supplementary file 14 — Supplementary information, Table S4 [file 41422_2024_1034_MOESM14_ESM.pdf]

Table S4. The impact of absolute gene expression values and model architecture on the performance of multiple downstream tasks. Geneformer and GeneCompass were pre-trained using the same full human dataset (~55 millions) and further finetuned on multiple downstream tasks, including cell type annotation, dosage sensitive TF classification, GRN inference, drug dose response prediction, and gene expression profiling. In the fine-tuning stage, 5-fold cross validation was performed. The best results for each task are marked in red.

Method		Cell type annotation hMS		Cell type annotation hLiver		Dosage sensitive TF classification	GRN inference	Drug dose response	Gene expression profiling
		Macro-f1	Accuracy	Macro-f1	Accuracy	AUC	AUPRC	R2	Rmse
Geneformer*		0.694±0.036	0.839±0.021	0.723±0.014	0.796±0.006	0.900±0.016	0.111±0.012	0.833±0.006	2.01084
GeneCompass	baseline (id+value)	0.726±0.016	0.835±0.011	0.725±0.007	0.810±0.004	0.916±0.029	0.114±0.009	0.797±0.004	2.00955
	baseline + all prior	0.748±0.034	0.856±0.003	0.746±0.008	0.817±0.008	0.950±0.023	0.120±0.005	0.877±0.002	2.00944

* indicated the model was retrained with the same corpus as GeneCompass.
