# Supplementary material for: GeneCompass: deciphering universal gene regulatory mechanisms with a knowledge-informed cross-species foundation model
Source: Cell Res. 2024 Oct 8;34(12):830–45. doi: 10.1038/s41422-024-01034-y (PMC11615217; doi:10.1038/s41422-024-01034-y)
Supplement: Supplementary file 15 — Supplementary information, Table S5 [file 41422_2024_1034_MOESM15_ESM.pdf]

Table S5. Comparison of GRN inference performance among different TFs. The table presented the performances for each transcription factor in GRN inference task, AUPRC (Area Under the Precision-Recall Curve) was applied as the metric. The transcription factors listed in the 'TF' column included OTX2, TFAP2A, MXI1, EOMES, and others, with their respective AUPRC values ranged approximately from 0.110 to 0.130.

TF	AUPRC	TF	AUPRC
OTX2	0.131127747	EZH2	0.111991423
TFAP2A	0.130459267	TCF4	0.111837519
MXI1	0.129482609	AR	0.111817551
EOMES	0.129023307	GATA2	0.111753555
NANOG	0.123756216	ZNF207	0.111738601
FOXP1	0.123517035	GATA6	0.111621514
SOX2	0.122404809	BCL11A	0.111599204
HAND1	0.121830914	SOX17	0.111456163
TET2	0.121830868	KLF5	0.111422554
SMAD2	0.121827881	TP53	0.111380449
MLLT3	0.121812269	FOXH1	0.111343831
UBTF	0.121810897	POU5F1	0.111220223
HAND2	0.121740616	STAT3	0.111205415
PRDM1	0.121622594	TRIM28	0.11107479
LEF1	0.121490346	SNAI2	0.111046344
CTNNB1	0.121452498		
GATA3	0.121358136		
GATA4	0.121351383		
ZFP42	0.121285386		
JUND	0.121034673		
TEAD4	0.114278439		
EGR1	0.113040507		
